# Metabolite Identification Data in Drug Discovery, Part 1: Data Generation and Trend Analysis

**DOI:** 10.1021/acs.molpharmaceut.5c00738

**Published:** 2025-10-14

**Authors:** Marie Ahlqvist, Isabella Bonner Karlsson, Anja Ekdahl, Cecilia Ericsson, Ulrik Jurva, Filip Miljković, Ya Chen, Susanne Winiwarter

**Affiliations:** † Drug Metabolism and Pharmacokinetics, Research and Early Development, Cardiovascular, Renal and Metabolism (CVRM), BioPharmaceuticals R&D, 468087 AstraZeneca, Pepparedsleden 1, 431 83 Mölndal, Sweden; ‡ Medicinal Chemistry, Research and Early Development, Cardiovascular, Renal and Metabolism (CVRM), BioPharmaceuticals R&D, 27258 AstraZeneca, Pepparedsleden 1, 431 83 Mölndal, Sweden; § Department of Pharmaceutical Sciences, Division of Pharmaceutical Chemistry, Faculty of Life Sciences, University of Vienna, Josef-Holaubek-Platz 2, 1090 Vienna, Austria

**Keywords:** metabolite identification, drug metabolism, LC-MS, data sharing, drug discovery

## Abstract

In drug discovery, metabolite identification data are used to identify metabolic soft spots in research molecules to facilitate reduced metabolism in subsequently designed compounds. In addition, knowledge about exact metabolite structures enables the assessment of risks associated with active, reactive, or toxic metabolites. In the present work, we exemplify how metabolite identification data are generated and utilized at AstraZeneca. We share metabolite transformation scheme data derived from incubations in human hepatocytes for a set of 120 compounds. Comparison with other in-house generated metabolite identification data, both in terms of chemical space analysis and *in vitro* properties, is performed, including the characterization of observed metabolic pathways. For selected compounds, the correlation between *in vitro* and *in vivo* metabolite data in animal species is provided. Finally, the usage of shared metabolite identification data for drug metabolism prediction using machine learning and artificial intelligence approaches is discussed.

## Graphical abstract

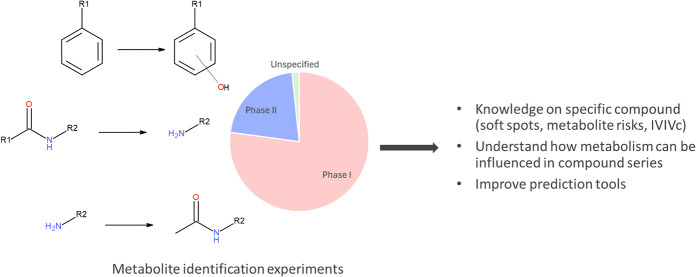

## Introduction

Drug metabolism studies are crucial for determining the pharmacokinetics (PK) and pharmacodynamics of drug molecules.^1–3^ In addition, incorporating biotransformation studies early in drug design is essential for developing novel drug candidates.^4,5^ For example, identifying the metabolic soft spots of lead molecules allows for tailoring molecular design toward compounds with reduced metabolic clearance.^6^ This is expected to lead to better overall PK properties and can also result in a decreased risk of forming metabolites that are reactive, toxic, or have drug–drug interaction liabilities.^7,8^ In addition, metabolism by cytochrome P450 (CYP) and other enzymes typically results in metabolites that are closely related to the original drug but display reduced lipophilicity.^9^ Sometimes, such metabolites can be utilized as a superior chemical foundation for lead optimization compared to their starting point.^10^

Metabolite identification (MetID) experiments are performed on samples from biological systems, such as *in vitro* hepatocyte incubations or samples from *in vivo* studies. The parent compound and formed metabolites are first separated by liquid chromatography (LC), which is followed by structural elucidation of the individual compounds using mass spectrometry (MS). In the absence of any radiolabel, MS peak areas cannot be used to exactly quantify the abundance of metabolites, since correct quantification requires the availability of synthesized standards and the generation of standard curves.^11,12^ In early discovery, usually no standard is available, but the peak areas are used for semiquantification to identify the major metabolite(s) and thereby the most important soft spot. The risk with this assessment is that the ionization efficiency of the parent compound and formed metabolites can differ significantly, and therefore, the assumption that the metabolite with the largest peak area is the major metabolite can simply be wrong. Please also note the differences between *in vitro* and *in vivo* samples for MetID. *In vitro* samples originate from “closed systems,” which continuously form primary or secondary metabolites without any other elimination processes and are dominated by the formation rates of the metabolites. In contrast, *in vivo* samples are taken from an “open system,” where the drug and its subsequent metabolites are eliminated not only by metabolism but also by other processes, such as active excretion by various transporters, i.e., plasma profiles are a product of formation and elimination rates of the metabolites. Thus, both the quantity and identity of the metabolites can differ between *in vitro* and *in vivo*.

Advances in high-resolution mass spectrometry (HRMS) have improved the detection of drug-related metabolites at trace concentrations. The challenge now lies in converting large amounts of raw data into useful insights for drug development. There are several available postexperimental MetID tools (MetaboLynx,^13^ CompoundDiscoverer,^14^ MetabolitePilot,^15^ MetaboScape,^16^ and MassMetaSite^17–20^ ) that can support the interpretation of raw data, hence increasing the number of MetID experiments that can be processed.

Furthermore, several software packages have been developed to predict the sites of metabolism (SoMs),^21–23^ also known as metabolic soft spots, or to suggest likely metabolites.^24,25^ Rule-based prediction methods use empirically derived rules from known metabolic reactions to predict SoMs. These rules are typically compiled into databases, from which the software extracts the relevant rule for the given chemical structure to determine likely SoMs and—if defined—possible metabolite structures (e.g., Meteor Nexus^26^ and BioTransformer^24^). Machine learning (ML) models, on the other hand, are trained on large data sets of known metabolic reactions to predict SoMs. These models learn patterns and relationships within the encoded SoM data that can be applied to new compounds (e.g., XenoSite,^22^ FAME 3,^21^ and MetaScore^27^). A more mechanistic approach is taken by SMARTCyp,^28^ a software that considers atom reactivity and steric effects to predict sites of CYP metabolism. Docking-based approaches use three-dimensional structural information to predict how drug molecules interact with metabolic enzymes such as members of the CYP family.^29^ Docking is often combined with other methods. IDSite^30^ is an example where an additional calculation step to estimate the reactivity of specific atoms completes the analysis. Another three-dimensional-based method, MetaSite^31^ predicts potential SoMs by aligning ligand structures to GRID molecular fields, which encode a fingerprint of the enzyme’s active site. Sufficiently reliable MetID prediction tools will make it possible to perform *in silico* MetID, i.e., enable estimates of likely soft spots in molecules or even define potential metabolites before a compound is actually made. However, more MetID data need to become publicly available to improve present prediction tools. Also, a large amount of drug metabolism data is stored in formats that are not easily accessible by machines. As a result, most MetID data must be converted, interpreted, and curated before they can be used to develop *in silico* approaches.

In this article, we share human *in vitro* metabolite schemes from hepatocyte incubations for 120 compounds from the AstraZeneca experimental MetID database. We discuss general data trends observed in comparison with other in-house generated MetID data, including chemical space and *in vitro* property analyses. In addition, we evaluate specific metabolism routes observed in the data, as well as the correlation between *in vitro* and *in vivo* MetID experiments performed in different animal species for selected compounds. In our view, sharing MetID data is critical for building effective software tools, utilizing ML and artificial intelligence (AI) approaches, that can reliably predict SoMs and metabolite structures of novel molecules of interest for drug discovery. Collaborative data sharing enhances both the quality and the quantity of data available for such model-building endeavors. Hereby, we invite others to join us in contributing additional proprietary experimental MetID data to the public domain, thus aiding the collaborative nature of open science and AI toward advancing drug metabolism prediction.^32^

## Methods and Materials

### Chemicals and Reagents

All investigated compounds, including the control compounds albendazole and dextromethorphan, were obtained from Compound Management (AstraZeneca, Gothenburg, Sweden). Acetonitrile (ACN) and methanol of high-performance liquid chromatography (HPLC) or LC/MS grade from Fisher Scientific, dimethyl sulfoxide (DMSO) from Sigma-Aldrich, formic acid (FA) of HPLC grade from Acros Organics, L-15 Leibovitz buffer (no phenol red) with l-glutamine (Gibco 21083–027), and cell counting solution Casyton (Roche Innovatis AG) were used. Water was purified by using an in-house Milli-Q water purification system.

### Typical Equipment

Tecan Freedom Evo robot, plate shaker Variomag Teleshake 70 with TEC Control 485. Tecan 100 μL red tips; NUNC 1 mL 96-deep-well PP Plate Natural (260252, Thermo Scientific); conical NUNC plates 0.45 mL/well (249944, Thermo Scientific); microwell lid (263339, Thermo Scientific); NUNC 96-well caps natural (276002, Thermo Scientific); Eppendorf pipettes; Rainin multipipettes; Eppendorf Easypet; vacuset; Grant water bath; Sigma Centrifuge 4K15; and Casy Innovatis cell counter.

### Hepatocyte Incubation

Cryopreserved, pooled primary human, dog, and rat hepatocytes were obtained from BioIVT (previously Celsis IVT). L-15 Leibovitz buffer was placed in a water bath to reach 37 °C. Cryopreserved hepatocytes were transferred from a −150 °C freezer on dry ice and immediately immersed in a preheated water bath kept at 37 °C for thawing. When only a small ice crystal could be seen, the content was emptied into a 50 mL falcon tube filled with warm buffer (maximum four vials/falcon tube). The suspension was centrifuged at room temperature for 3 min at 50 g. The supernatant was removed, the pellet was resuspended in a small volume of buffer, and the falcon tube was refilled with buffer, followed by another centrifugation to wash away any remaining storage buffer. Thereafter, the supernatant was removed, and the pellet was dissolved in a small volume of buffer and diluted to a concentration of about 3–5 million cells/mL. After counting the cells using a Casy cell counter, the suspension was diluted to 1 million viable cells/mL and kept at room temperature until use. The viability cutoff was 80%.

An aliquot of 245 μL of hepatocyte suspension was added to a round-bottomed 96-deep-well plate using a manual multipipette. The deep-well plate was preincubated for 15 min at 37 °C and 13 Hz. Substrate solutions of the assay compounds were prepared by the robot in a 96-well plate as follows: 4 μL of 10 mM DMSO stock solution, followed by the addition of 96 μL of ACN:water (1:1, v:v) and mixing by shaking. 50 μL from each well were transferred to and combined in a new plate, resulting in 100 μL per well and a dilution of 1:1.

The reaction was started by the addition of 5 μL of 200 μM substrate solution to the preheated hepatocyte suspension, resulting in a final substrate concentration of 4 μM (0.04% DMSO, <0.5% ACN). The incubation was continued at 37 °C and 13 Hz. At each time point (0, 40, and 120 min), a 50 μL sample was taken out and quenched in 200 μL of cold ACN:methanol (1:1, v:v), and the stopped plates were centrifuged for 20 min (set at 4 °C and 4000 g). The supernatant was diluted by taking 50 μL and mixing it with 100 μL of water. Incubations of the positive controls, albendazole and dextromethorphan, were performed in parallel as described above.

### MetID on *In Vivo* Samples

Plasma and urine samples (if available) were obtained from dogs (beagles) and rats (Han Wistar) *in vivo* studies performed in accordance with relevant guidelines and regulations and approved by the local Animal Research Ethics Board of Gothenburg, Sweden (rat: 190–2010, approved 2010–08–30; dog: 132–2010, approved 2010–05–18). The workup procedure could vary somewhat depending on the compound and dose, but the general procedure was as follows: Two volumes of ACN/methanol (1:1, v:v) were added to the plasma samples and vortex-mixed to precipitate the proteins. Thereafter, the samples were centrifuged for 10 min (at 10,000 *g* and 4 °C), and the supernatants were diluted with one volume of water. The urine samples were diluted with one volume of ACN/methanol (1:1, v:v) and centrifuged for 10 min (at 10,000 *g* and 4 °C), followed by a 1:1 dilution with water.

### LC-MS Analysis for Metabolite Profiling

Structural characterization of metabolites and metabolite profiling were performed using the Waters Acquity ultraperformance liquid chromatography (UPLC) system fitted to a Waters High-Resolution Mass Spectrometer (MS) (Xevo G2-S, Synapt G2-Si, or a Synapt XS MS). The Waters MS systems, equipped with an electrospray ionization (ESI) source, were operated in positive-ion sensitivity mode over a mass range *m*/*z* 50–1200 and in negative-ion sensitivity mode over a mass range *m*/*z* of 50–1200.

The reversed-phase gradient elution was performed on an Acquity U­(H)­PLC BEH C18 column (2.1 × 100 mm, 1.7 μm, Waters) set at 45 °C. The mobile phases consisted of 0.1% FA in water (A) and ACN (B). The gradient used was as follows: 5% B at 0 min, increased to 40% B at 4.0 min, and further increased to 95% B at 6.5 min. The solvent composition was held at 95% B for 1.0 min and then decreased to 5% B at 7.5 min, followed by re-equilibration at 5% B for 0.5 min. The injection volume was 2 μL. The MS settings were optimized for the respective MS instrument. For example, for Waters Synapt G2-Si and Synapt XS instruments, the following standard settings were used: MSe data was typically generated over *m*/*z* range of 100–1200 (low-energy function) and *m*/*z* 50–1200 (high-energy function). A scan time of 0.1 s was used, and data were acquired in centroid mode. Collision energy was set to “off” for the low-energy function, whereas in the high-energy MSe functions, the collision energies were typically ramped from 15 to 35 V in the trap cell, with constant collision energies of 20 V in their transfer cells. Cone voltage was set to 25 V for both low- and high-energy functions. For dedicated MS/MS, the *m*/*z* of the molecular ion was selected in the quadrupole, and the mass range for fragmentation analysis was typically set between 50 and 1200. The collision energies were either the same as those stated above for MSe data generation or optimized for a specific metabolite. Tune parameters for all MS analyses consisted of a capillary energy of 0.5 kV, sampling cone energy of 40 V, source temperature of 150 °C, desolvation temperature of 550 °C, cone gas flow of 0 L/h, desolvation gas flow of 1200 L/h, and nebulizer pressure of 6.5 bar.

### Metabolite Identification

Metabolite identification was conducted by processing the generated data using the MetaboLynx XS browser (Waters),^13^ MassMetaSite^17^ and the web interface ONIRO^33^ (Mass Analytica). A list of common phase I and II transformations (see Table S1) was used for the identification of the expected metabolites, and potential unexpected metabolites within the mass defect filtering region were also suggested by the software. Accurate masses of metabolites were determined from the protonated molecules in the positive ESI time-of-flight MS (ESI–TOF–MS) mode and from the deprotonated molecules in the negative ESI–TOF–MS mode. The mass error for each proposed metabolite and fragment ion structure was <5 ppm. For those metabolites where no MS/MS data could be acquired, identification was based on the accurate mass of the molecular ion and the fragmentation seen in the MSe spectrum. Relative amounts of detected metabolites were calculated as % of the total MS area by dividing the MS peak area for the metabolite (or parent compound) by the combined MS peak area for the parent compound and all identified metabolites.

### Additional *In Vitro* ADME Properties

*In vitro* experimental data were generated utilizing routine high-throughput screening assays with at least 96-well plates, starting from a 10 mM DMSO stock solution of the query compounds as delivered by Compound Management. For all assays, appropriate control compounds were utilized in each run.

**Log*D*** was measured using the traditional shake-flask method, with up to ten compounds pooled together. The compounds were dissolved in 10 mM sodium phosphate buffer adjusted to pH 7.4 and an octanol phase (both phases were mixed for 2 h at room temperature). The concentrations in both phases were analyzed by liquid chromatography and quantitative tandem mass spectrometry (LC-MS/MS), and distribution coefficients were determined.^34^

**Solubility** was measured as the thermodynamic solubility using a shake-flask approach. Here, DMSO was evaporated from the compound stock solution as the first step. The “dried” compounds were then placed into 10 mM sodium phosphate buffer, adjusted to pH 7.4, and equilibrated at 25 °C for 24 h. The samples were centrifuged, and the solution was analyzed by LC-MS/MS to determine the amount of compound in solution.^35^

**Metabolic stability in human hepatocytes** (HH CLint) was measured by incubating the cells with the test compound at 37 °C for 2 h. An aliquot of the mixture was taken at typically nine defined time points, and the metabolic reaction was stopped by adding ACN. The samples were centrifuged, and the supernatant was analyzed via LC-MS/MS. Intrinsic clearance (CLint) was calculated from the disappearance rate of the parent compound.^36,37^

**Metabolic stability in human liver microsomes** (HLM CLint) used a similar procedure: here, query compounds were incubated with human liver microsomes and nicotinamide adenine dinucleotide phosphate (NADPH) in a phosphate buffer solution at pH 7.4 and 37 °C for 30 min. Aliquots of the incubation mixture were drawn at six defined time points, the metabolic reaction was stopped with ACN, the samples were centrifuged, and the supernatant was analyzed using LC-MS/MS. The CLint was calculated from the compound disappearance rate.^36,37^

**Human plasma protein binding** (HPB) was measured in 100% plasma using equilibrium dialysis. Up to ten compounds were pooled together. The dialysis time was 18 h, and a CO_2_ incubator was used for pH control. Plasma and buffer samples were analyzed by LC-MS/MS, and both the fraction unbound (fu, also given as % free) and the apparent affinity constant *K* (=[1 – fu]/fu) were determined.^38,39^

**Fraction of unbound drug in the rat hepatocyte incubation** (RH fuinc) was determined by equilibrium analysis. Rat hepatocytes were incubated at 37 °C with the CYP inhibitor 1-aminobenzotriazole (1-ABT) at 1 mM for 1 h. After the addition of salicylamide (1.5 mM) and an additional 5 min of incubation, test compounds were added (1 μM), and the mixed solution was transferred to the rapid equilibrium dialysis (RED) device (Thermo Fischer Scientific Inc., Rockford, USA) and dialyzed against media buffer for 4 h at 37 °C. Samples from both sides were analyzed by LC-MS/MS, and the fraction unbound in the incubation was determined, as well as the corresponding apparent affinity constant *K* (=[1 – fu]/fu).^40^

### Data Sets

The AstraZeneca experimental database was queried for compounds with available human hepatocyte metabolite schemes generated at the Gothenburg site, resulting in 2042 compounds. From this collection, 120 compounds were selected for data sharing (“*120 MetID compounds*”), whereas the remaining 1922 molecules were used as a reference for the chemical space and *in vitro* parameter comparison analyses (“*Other MetID compounds*”).

### Chemical Space Analysis

Nonstereoisomeric Simplified Molecular Input Line Entry System (SMILES) representations were generated from internally canonicalized isomeric SMILES for all compounds using RDKit.^41^ SMILES codes for the *120 MetID compounds* can be found in Table S2. For each compound, 15 physicochemical descriptors were calculated: number of nitrogen atoms, number of oxygen atoms, number of chiral centers, molecular weight, number of heavy atoms, number of hydrogen bond acceptors, number of hydrogen bond donors, log*P*, topological polar surface area, number of aromatic atoms, number of rings, fraction of Csp^3^ atoms, number of sulfur atoms, number of halogen atoms, and molar refractivity. Chemical space analysis was performed using Uniform Manifold Approximation and Projection (UMAP)^42^ in Python with default settings (umap-learn^43^ package). In addition, we extracted a corresponding Bemis-Murcko scaffold^44^ for each compound’s nonstereoisomeric SMILES using RDKit^41^ to further investigate the chemical space overlap between the two compound groups at the molecular framework level.

### *In Vitro* Parameter Analysis

Experimental values for Log*D*, solubility, metabolic stability (HH CLint, HLM CLint), and fraction of unbound drug (HPB, RH fuinc) were also extracted from the AstraZeneca collection (see section “Additional *In Vitro* ADME Properties”). If several replicates existed for the same molecule, then an average value was considered. The average values (*x̅*) were retained if they conformed to the experimental dynamic range requirement for each *in vitro* parameter. Else, they were capped at either the lower or the upper bound if beyond the range. The experimental dynamic ranges for each *in vitro* parameter were as follows : Log*D* (−1.5 ≤ *x̅* ≤ 4.5), solubility (0.1 μM ≤ *x̅* ≤ 1500 μM), HH CLint (1 μL/min/1E6 ≤ *x̅* ≤ 300 μL/min/1E6), HLM CLint (3 μL/min/mg ≤ *x̅* ≤ 300 μL/min/mg), HPB (Log*K*) (−0.95 ≤ *x̅* ≤ 3.30), and RH fuinc (Log*K*) (−0.95 ≤ *x̅* ≤ 3.30). Averaged solubility, HH CLint, and HLM CLint measurements were converted to corresponding decadic logarithm values to enable easier comparison of value distributions. The distribution of *in vitro* parameter values was then compared between 120 compounds designated for data sharing and other molecules tested in the historical MetID experiments.

## Results and Discussion

### Metabolite Identification Controls

The hepatocyte capacity for phase I and phase II metabolism was evaluated by monitoring the formation of relevant metabolites of the positive controls, albendazole and dextromethorphan (Figure 1). For albendazole, the monooxygenation to albendazole sulfoxide, metabolized via CYP1A2, 3A4, and 2J2, as well as by flavin-containing monooxygenase (FMO), was monitored.^45,46^ For dextromethorphan, the *O*-demethylation via CYP2D6, the *N*-demethylation via CYP3A4, and the *O*-glucuronidation via uridine 5′-diphospho-glucuronosyltransferase (UGT) were monitored.^47,48^ If the turnover of the control compounds was deemed too low or if the expected metabolites were not observed, the experiment was not approved.

**Figure 1:**
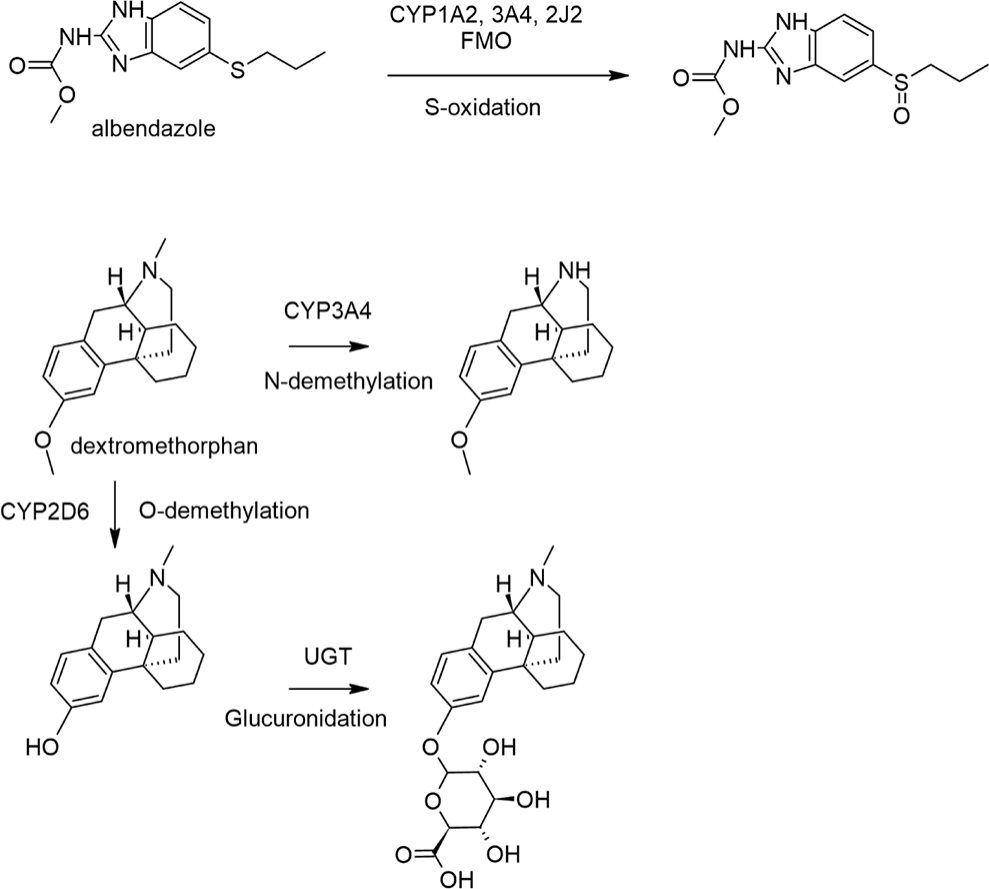
Metabolite schemes of albendazole and dextromethorphan. Shown are metabolite schemes of two positive controls, albendazole (top) and dextromethorphan (bottom), alongside their major metabolites and enzymes coordinating biotransformation.

### Chemical Space and *In Vitro* Property Analyses

Figure 2 displays the UMAP-generated chemical space visualization of a total of 2042 compounds that were historically evaluated at AstraZeneca Gothenburg using *in vitro* MetID experiments (human hepatocytes). The publicly shared *120 MetID compounds* (yellow circles) are nearly evenly distributed across the major compound clusters formed by the remaining 1922 internal compounds (*Other MetID compounds*, blue crosses). Several smaller clusters remain distinct from the distribution of the *120 MetID compounds*, possibly because they originate from drug projects that explored different compound chemistries. Moreover, we generated Bemis-Murcko^44^ scaffolds for both groups of molecules using RDKit^41^ in order to evaluate their scaffold composition and overlap. We identified 77 distinct scaffolds for the publicly shared data (*120 MetID compounds*), while 1138 scaffolds were obtained in the remaining internal data set (*Other MetID compounds*). At the intersection, 40 shared Bemis-Murcko scaffolds were identified, which were contained in 79 compounds from the *120 MetID compounds* and 202 compounds from the internal reference data set. This demonstrates that the majority of the compounds shared in this work (∼66%) had Bemis-Murcko scaffolds that were identical to those found in the remaining internal data. Both chemical space analysis and scaffold overlap show that the *120 MetID compounds* are representative of the chemical space of the compounds tested in MetID experiments at AstraZeneca. In addition, the 120-compound set contains a diverse set of chemical scaffolds (1.56 compounds per scaffold) that should be of interest to the wider scientific community focusing on drug metabolism studies.

**Figure 2:**
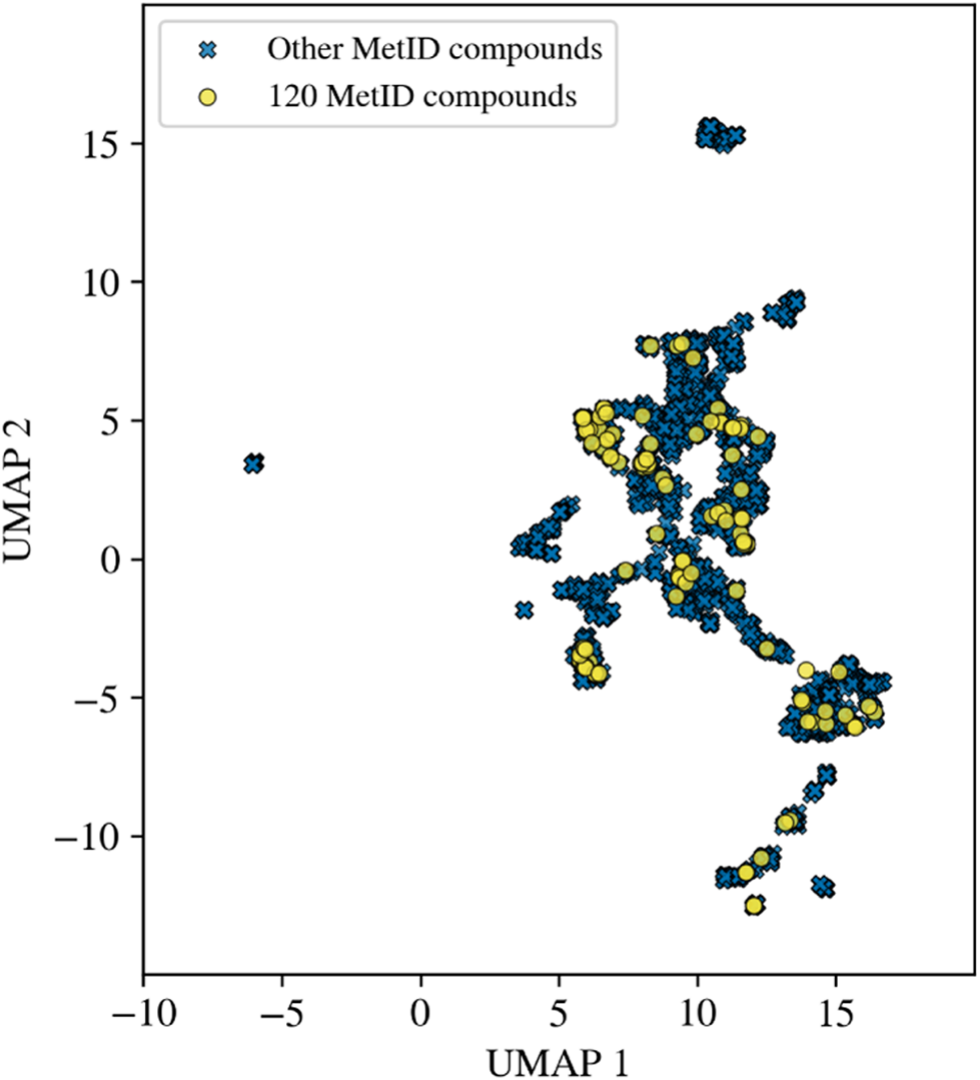
Chemical space comparison between shared and internal AstraZeneca MetID compounds. The UMAP plot compares the chemical space covered by *120 MetID compounds* (yellow circles) and *Other MetID* *compounds* (blue crosses, *n* = 1922), based on 15 physicochemical descriptors (see Methods for details).

Next, we compared the distribution of six different *in vitro* physicochemical and ADME properties measured at AstraZeneca for the *120 MetID compounds* and the internal reference set. These *in vitro* properties were Log*D*, solubility, HH CLint, HLM CLint, HPB (Log*K*), and RH fuinc (Log*K*). Statistics regarding the number of replicates for each *in vitro* property across 2042 compounds (*120 MetID* *compounds* and 1922 *Other MetID compounds*) are shown in Table 1.

**Table 1:** Number of Replicates per *In Vitro* Parameter for 2042 Compounds^a^

#Replicates	LogD	Solubility	HH CLint	HLM CLint	HPB	RH fuinc
0	573 (28.06%)	202 (9.89%)	269 (13.17%)	163 (7.98%)	250 (12.24%)	1311 (64.20%)
1	936 (45.84%)	1209 (59.21%)	1124 (55.05%)	1264 (61.90%)	1100 (53.87%)	95 (4.65%)
2	302 (14.79%)	365 (17.87%)	380 (18.61%)	349 (17.09%)	398 (19.49%)	351 (17.19%)
≥3	231 (11.31%)	266 (13.03%)	269 (13.17%)	266 (13.03%)	294 (14.40%)	285 (13.96%)

a For each *in vitro* parameter, the number of compounds (including the percentage) containing one, two, or at least three replicates is shown. In addition, the number of compounds with no measurements for a particular parameter (#Replicates = 0) is displayed.

Median values for *120 MetID compounds* were similar or slightly different from those of *Other MetID compounds*: Log*D* (2.52 to 2.39), solubility (μM, log-transformed: 1.95 vs 1.97), HH CLint (μL/min/million cells, log-transformed: 0.78 vs 0.86), HLM CLint (μL/min/mg protein, log-transformed: 1.08 vs 1.26), HPB Log*K* (0.94 vs 1.27), and RH fuinc Log*K* (−0.16 vs −0.03) (Figure 3). On average, a majority of the compounds in both groups had Log*D* values around 2 (typical of orally bioavailable small-molecule drugs^49,50^ ) high solubility, were stable in human hepatocytes and microsomes, and had low percent free protein binding in both human and rat. *Other MetID compounds* included a significant number of compounds with higher clearance values and increased binding to proteins. The distribution range of *120 MetID compounds* fell well within the range of other internal compounds and was typical of molecules at various stages of preclinical development. This was expected since MetID experiments are typically performed when there is interest in gaining a better understanding of the metabolic pathways of the lead chemical series, including the safety implications of lead molecules and their metabolites.

**Figure 3:**
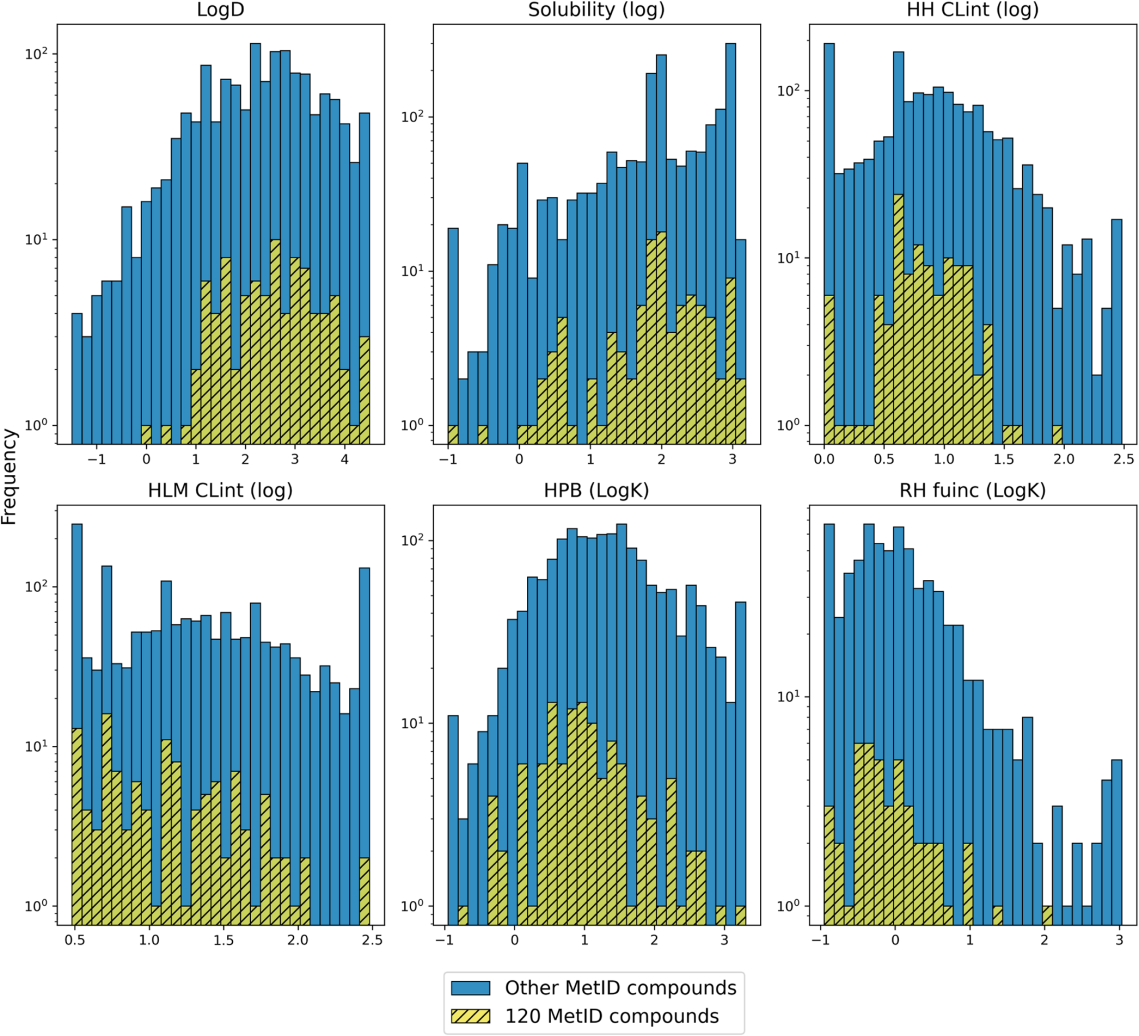
Comparison of the distribution of six *in vitro* properties for *120 MetID compounds* (yellow, hatched) and *Other MetID compounds* (blue, *n* = 1922). Shown are distribution histograms for six physicochemical and ADME properties (top, left to right: Log*D*, solubility (μM, log), HH CLint (μL/min/million cells, log); bottom, left to right: HLM CLint (μL/min/mg protein, log), HPB (Log*K*), and RH fuinc (Log*K*)). For each property, the value range is shown on the *x*-axis and on the *y*-axis frequency of compounds.

### Analysis of Metabolites and Covered Metabolic Pathways

For four of the *120 MetID compounds* (3%), no metabolites were identified. These compounds also showed low CLint values in the metabolic stability assays, falling below the lower limit of the dynamic assay range (see *In Vitro* Parameter Analysis). For the remaining 116 compounds, all metabolites were well-defined in 33% of the compounds, whereas at least one Markush structure was found in 67% of the compounds. Altogether, 436 metabolites were described for these compounds, of which 40% contained at least one Markush structure.

While the aim of metabolite identification is to define the metabolites as accurately as possible, this task is not always easy to accomplish, since compounds behave differently in the mass spectrometer and the fragmentation pattern needs to be understood. If the compound series is well-known, the task to assign the exact metabolite structure is usually easier, as earlier experience will help. In addition, the question asked by the project affects the results. In early discovery, projects often want to know the major metabolite to understand the metabolic soft spot of the compound. This knowledge is needed quickly so that it can be fed into the next design cycle; hence, the metabolite identification work will focus on the major metabolites only. Later on, specific compounds will be more thoroughly investigated, and more time and effort can be put into identifying all metabolites. In addition, more abundant metabolites have a better chance of being identified.

In this data set, we cover both phase I and phase II metabolism pathways, with more phase I reactions seen than phase II metabolism (see Figure 4). The single most frequent pathway in this set appears to be aliphatic hydroxylation. Other phase I pathways include aromatic hydroxylation and various oxidations, *N*- and O-dealkylations, and hydrolysis reactions, such as ester and amide hydrolysis. In some cases, it was not possible to define the exact metabolite, e.g., one could not distinguish between aromatic or aliphatic hydroxylation, but it was evident that the biotransformation pathway was hydroxylation. Also, for ring-opening reactions, it was often not possible to identify the first reaction, since it could be due to either hydrolysis or oxygenation. We also see less common reactions, such as the reduction of ketones to alcohols, which, for one of the compound series, turns out to be quite common. The most common conjugation reaction in this data set is glucuronidation, which encompasses more than 50% of all phase II metabolism reactions. However, we also observe sulfation and glycine or cysteine conjugation. These findings, especially the most frequent phase I and phase II metabolism reactions identified, are in line with known enzyme abundance: CYP3A4/5, the most common CYP enzyme, usually leads to hydroxylations, whereas the most important phase II enzymes are UGTs, which result in glucuronidations.^51^

**Figure 4:**
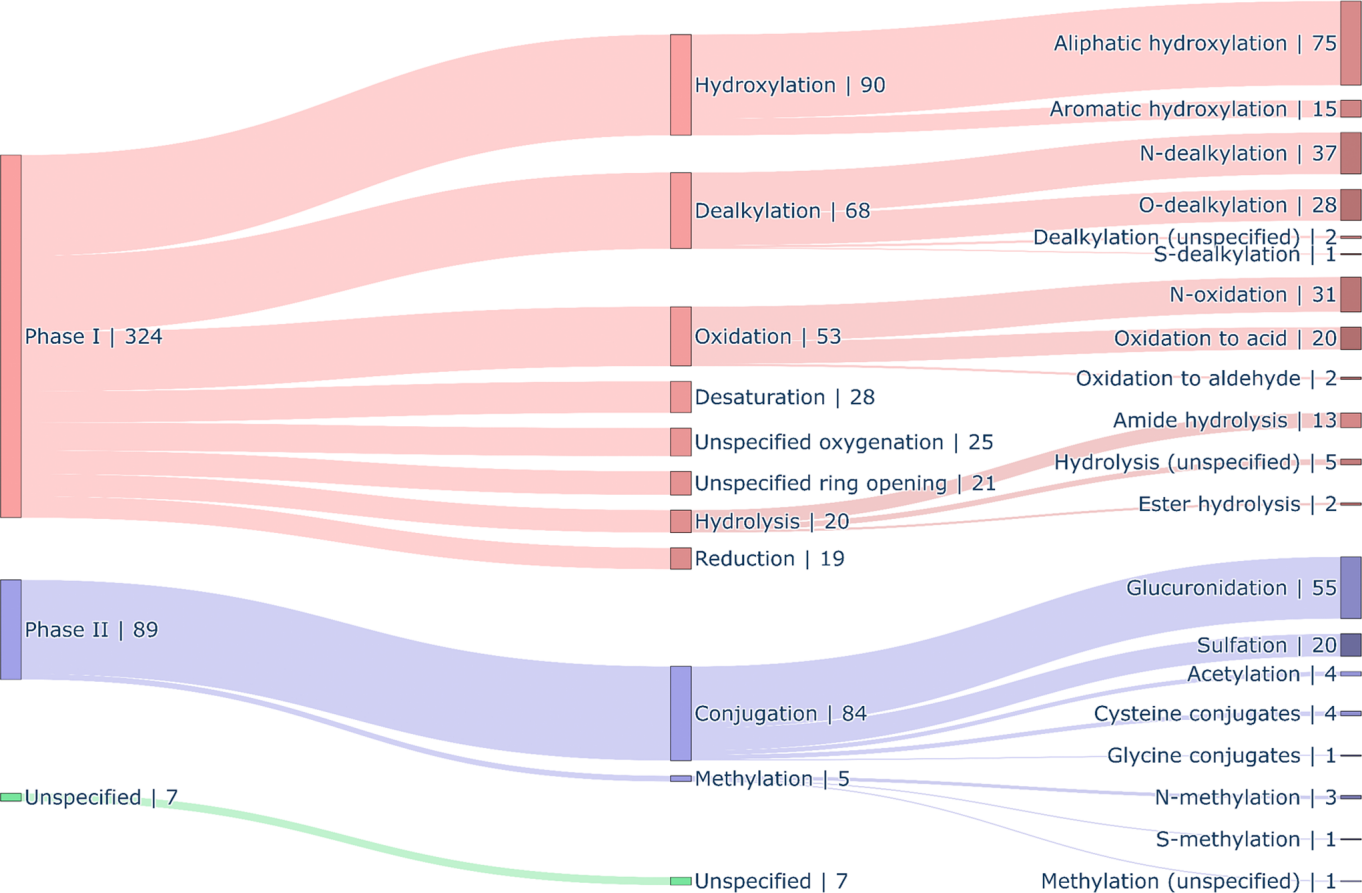
Illustration of metabolic pathways covered in the data set. Shown is a Sankey diagram of metabolic pathways associated with the MetID experiments of 120 compounds, considering the frequency of occurrence. Two major branches of metabolic pathways, namely phase I (red) and phase II (blue), are defined, each branching into more specific chemical transformations defined at two further hierarchical levels. An additional branch, termed Unspecified (green), was defined as well, where no clear metabolic pathway could be assigned. Each reaction is associated with the frequency of occurrence displayed by its name, and the value assigned to the reaction group is the sum of individual reactions or groups.

### Metabolite ID Scheme Comparison for Selected Matched Pairs

Next, we evaluated matched molecular pairs (MMPs) formed by 120 compounds, for which MetID data were shared, to identify chemical transformations that could explain differences in observed MetIDs for the generated pairs of molecules. An MMP can be defined as a pair of compounds that are distinguished by a small structural change at a single site.^52^ In total, we identified 34 MMPs within the AstraZeneca MMP database, generated using the in-house MCPairs tool,^53^ several interesting examples of which are displayed in Figures 5 and 6

**Figure 5:**
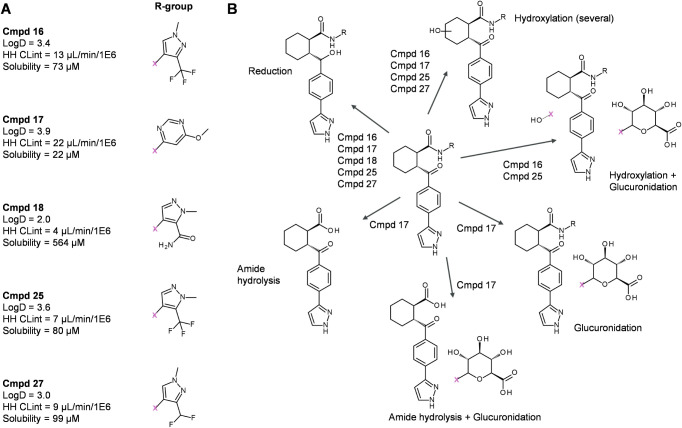
First example of matched molecular pairs with different metabolic transformations. Displayed are five compounds that differ in a single *R*-group, with specific residue and information on LogD, HH CLint, and solubility for each compound shown (A), and the summarized MetID schemes displaying the metabolic transformations and products (B).

**Figure 6:**
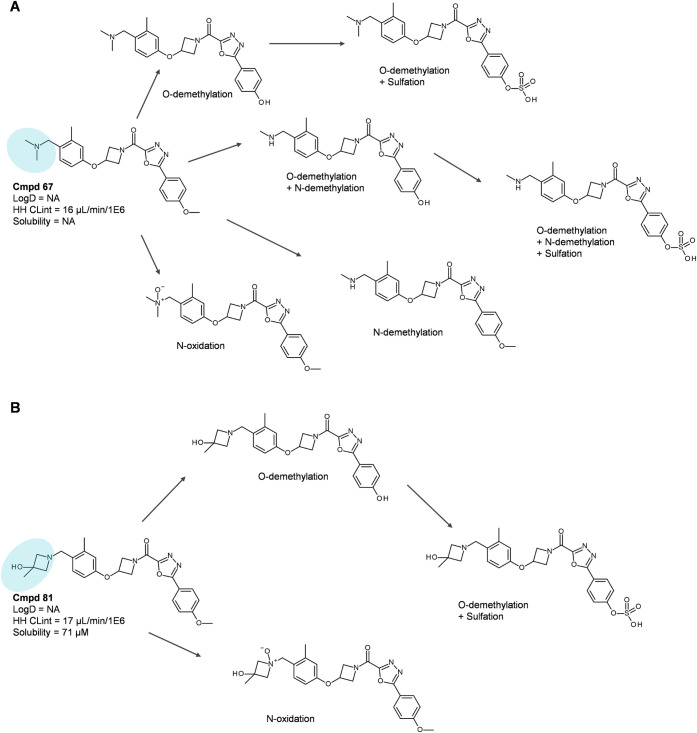
Second example of matched molecular pairs with different metabolic transformations. Shown are the MetID schemes for Cmpd 67 (A) and Cmpd 81 (B), displaying metabolic transformations and products. Differing molecular fragments are highlighted in blue, and Log*D*, HH CLint, and solubility values are provided for each compound (NA – “not available”).

Figure 5 shows five compounds in a series: Cmpd 16, Cmpd 17, Cmpd 18, Cmpd 25, and Cmpd 27. These compounds differ only in the upper aromatic residue (R-group depicted in Figure 5A). Cmpd 18 is the metabolically most stable compound, likely due to its lower lipophilicity. For this compound, the only metabolite identified was the alcohol formed after the reduction of a ketone. Cmpd 17, with the highest lipophilicity, is the least metabolically stable compound, and its metabolism is distributed across several metabolic pathways, including ketone reduction, aliphatic hydroxylations, *N*-glucuronidation, amide hydrolysis, and combinations thereof. For Cmpds 16, 25, and 27, amide hydrolysis was not detected, but otherwise the same metabolic pathways as for Cmpd 17 were seen. These three compounds also showed metabolic stability within the range between Cmpds 18 and 17. This compound series shows that even highly similar compounds can display different metabolic pathways and conversion rates. Metabolic stability correlated with the compounds’ lipophilicity, a major parameter usually considered during medicinal chemistry design, aiming at lower values to achieve molecules with improved metabolic stability within a chemical series.

In the second example, displayed in Figure 6 (with varying residues highlighted in blue), *N*- and *O*-demethylation are major metabolic pathways for Cmpd 67. Replacing the tertiary amine moiety with a hydroxy azetidine moiety in Cmpd 81 blocks the *N*-demethylation pathway, whereas the *O*-demethylation pathway remains the major metabolic route. Even though both compounds displayed equivalent metabolic stability in human hepatocytes, one can fine-tune elimination via the preferred metabolic pathway by protecting certain chemical moieties from being transformed. Careful design can thus lead to molecules that, for example, avoid biotransformation into reactive or potentially toxic metabolites.

### Comparing *In* *Vitro* and *In* *Vivo* Metabolite Schemes

Metabolite *in vitro*–*in vivo* correlation (IVIVc) was evaluated for the compounds for which *in vivo* data were available, mainly from routine rat and dog PK studies, by comparing metabolite profiles in hepatocytes with *in vivo* metabolites detected in plasma and/or urine. In general, good metabolite IVIVc was observed, and most of the major *in vivo* metabolites were formed in the hepatocytes. It is important to note that the comparisons are based on relative MS peak areas. Since no calibration curves or internal standards were utilized, and ionization efficiencies may differ significantly between the parent compound and its metabolites, these values should be considered only semiquantitative. Some of the compounds are discussed in more detail below.

*Cmpd 16.* The major metabolite (5%) in dog plasma was a hydroxylation of the cyclohexane ring (M1) (see Figure 7 and Table 2). This metabolite was not detected in dog hepatocytes, but two other hydroxylations of the cyclohexane ring (M3 and M4) were identified in dog hepatocytes. M3 and M4 were also observed in dog plasma, but at lower levels than M1 (M3 – 2%, M4 – 1%). In addition to M3 and M4, one more metabolite was seen in the dog hepatocytes: aromatic hydroxylation M6 (2%). Low levels (0.5%) of M6 were also detected in dog plasma. An amide hydrolysis (M10 – 1%) and an *N*-demethylation (M12 – 2%) were observed in dog plasma but not in dog hepatocytes.

**Table 2:** Detected Metabolites of Cmpd 16 and Cmpd 28^a^ ^b^

Compound name	Dog Plasma (Female)^c^	Dog Hepatocytes (Mixed^d^ gender)	Rat Plasma^e^ (Male)	Rat Plasma (Female)^e^	Rat Hepatocytes (Mixed^d^ gender)
**Cmpd 16**					
Parent	85	91	41	85	70
M1	5	NR	1	2	1
M3	2	2	45	6	8
M4	1	2	NR	1	1
M5	0.5	NR	NR	NR	9
M6	0.5	2	NR	NR	NR
M9	NR	NR	5	NR	NR
M10	1	NR	1	1	NR
M11	NR	NR	1	NR	NR
M12	2	NR	NR	2	NR
M13	0.5	NR	NR	NR	NR
M14	0.5	NR	NR	NR	NR
**Cmpd 28**					
Parent	34	79/6/5 *(three peaks)*	53/10/3 *(three peaks)*	63/9/4 *(three peaks)*	28/3 *(two peaks)*
M1	12	4	17	18	NR
M2	40	4	0.5	1	NR
M3	0.5	NR	3	1	5
M4	1	2	NR	NR	3
M5	2	NR	6	2	5
M7	5	NR	NR	NR	35
M8	NR	NR	3	1	NR
M9	NR	NR	NR	NR	9
M10	NR	NR	1	NR	NR
M11	0.5	NR	1	<1	NR
M12	NR	NR	1	1	NR

a The values are given as % of the total MS area of parent and identified metabolites in each system.

b NR – Not reported.

c Sampled at 3 h and 30 min.

d Incubated for 120 min.

e Sampled at 60 min for Cmpd 16 and at 4 h for Cmpd 28.

**Figure 7:**
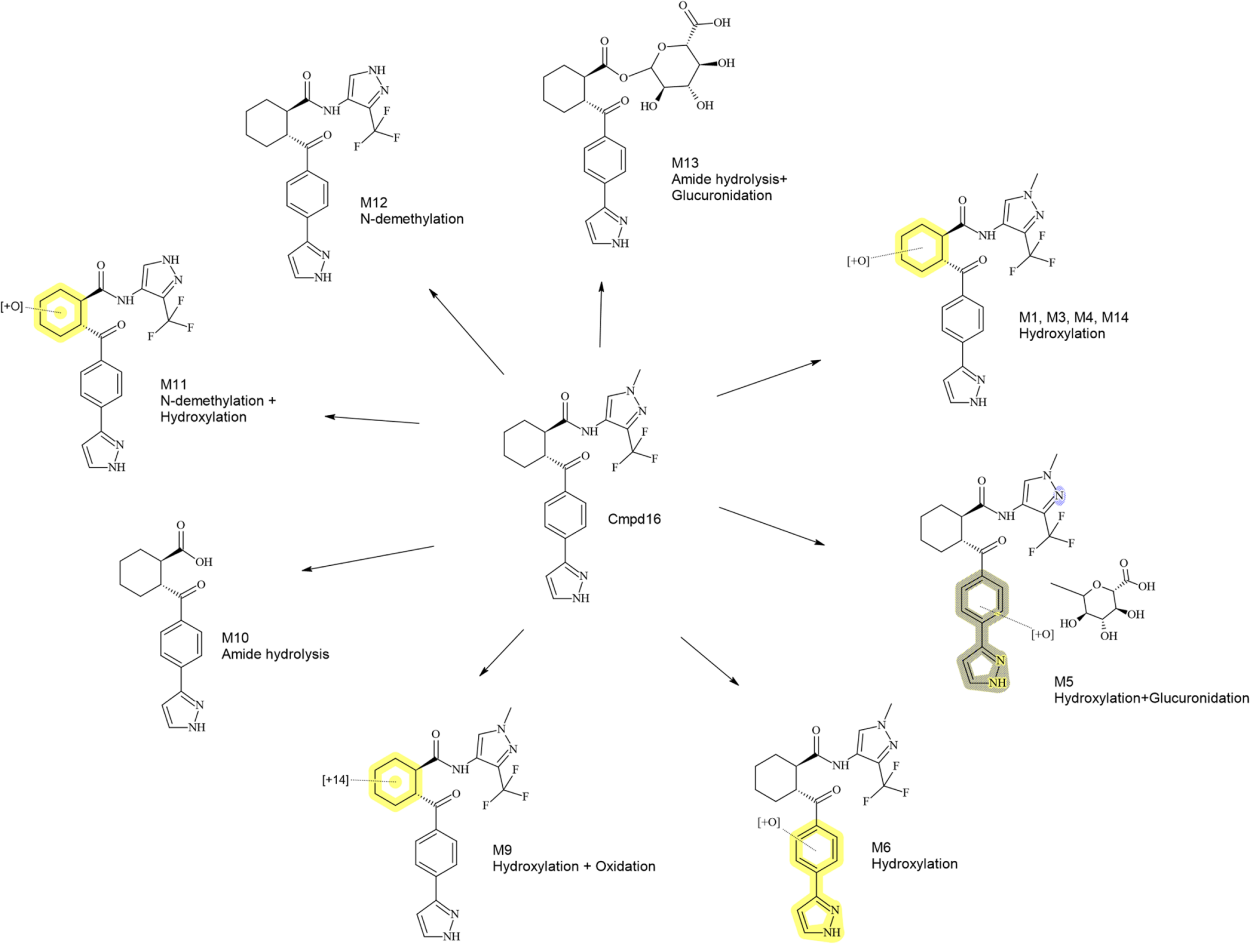
Metabolite profiling of Cmpd 16. Shown is a MetID scheme of Cmpd 16 together with its detected metabolites. Each metabolite is characterized by the observed transformation mechanism and the highlighted portion of the molecule where biotransformation takes place (in cases where exact conversion cannot be confidently assigned) or the exact chemical structure (where biotransformation assignment is certain). Detailed information about the species/system in which each metabolite is formed can be found in Table 2.

Biotransformation of Cmpd 16 was also investigated in the rat. In rat hepatocytes, three hydroxylations of the cyclohexane ring (M1, M3, and M4) were detected, with M3 being the major of the three at 8%. A fourth metabolite, involving hydroxylation and glucuronidation (M5), was also reported from rat hepatocyte incubation. Cmpd 16 was assessed *in vivo* in both male and female rats (same strain, Han Wistar), and substantial differences were seen, at least quantitatively, between the genders. One hour after oral administration, the parent compound constituted 85% of female rat plasma, whereas it only amounted to 41% in male rat plasma. This difference in the amount of parent compound is mainly due to large differences in the detected levels of the hydroxylation M3 between male and female rats. Although M3 is the major circulating metabolite in female rats at 6%, it constitutes 45% in male rat plasma. Similarly, as in the dog, amide hydrolysis (M10) and *N*-demethylation (M11, also hydroxylated, in male, and M12 in female rats) were observed in rats *in vivo* but not *in vitro*. Considering the comparatively low levels of these metabolites (1–2%) in dog and rat plasma, however, it cannot be excluded that they are formed in hepatocytes but at levels below the detection limits.

*Cmpd 28.* In dog plasma, a total of seven metabolites were reported, with amide hydrolysis (M1) and amide hydrolysis followed by glucuronidation (M2) as the two major metabolites at 12% and 40%, respectively (see Figure 8 and Table 3). In addition to M1 and M2, three different hydroxylations (M3–M5), one *N*-demethylation (M11), and a secondary metabolite (M7) were detected. M7, the carboxylic acid formed from hydroxylation M4, was the third largest *in vivo* metabolite at 5%. Three metabolites were reported from *in vitro* incubation in dog hepatocytes: M1 (4%), M2 (4%) and M4 (2%). Although the third largest circulating *in vivo* metabolite (M7) was not detected *in vitro*, the same metabolic pathway was still captured, as seen by the detection of M4, the precursor of M7. The metabolic conversion was slower *in vitro* than *in vivo*, but as the same metabolic pathways were dominant—i.e., amide hydrolysis (followed by glucuronidation) and hydroxylation of the methyl group on the pyrazole (M4)—metabolite IVIVc was deemed to be good in dog.

**Table 3:** Detected Metabolites of Cmpd 47, Cmpd 50, Cmpd 51, and Cmpd 55^a^ ^b^

	Parent	Glucuronide	Ring-opening and oxidation	Ring-opening and reduction	*N*-oxide
**Cmpd 47**					
Hepatocytes^c^	83	7	2	NR	1
Plasma^d^	5	69	17	4	4
Urine^e^	5	41	32	NR	3
**Cmpd 50**					
Hepatocytes^c^	73	18	4	3	1
Plasma^f^	>99	NR	NR	NR	NR
Urine^e^	10	41	19	3	NR
**Cmpd 51**					
Hepatocytes^c^	80	8	2	NR	1
Plasma^f^	20	57	17	3	NR
Urine^e^	22	35	32	NR	NR
**Cmpd 55**					
Hepatocytes^c^	97	0.2	0.7	NR	NR
Plasma^f^	49	5	10	2	NR
Urine^e^	61	10	8	2	NR

a The values are given as % of the total MS area of parent and identified metabolites in dog hepatocytes, plasma or urine.

b NR – Not reported.

c Incubated for 120 min.

d Sampled at 30 min.

e Sampled at 7–24 h.

f Sampled at 60 min.

**Figure 8:**
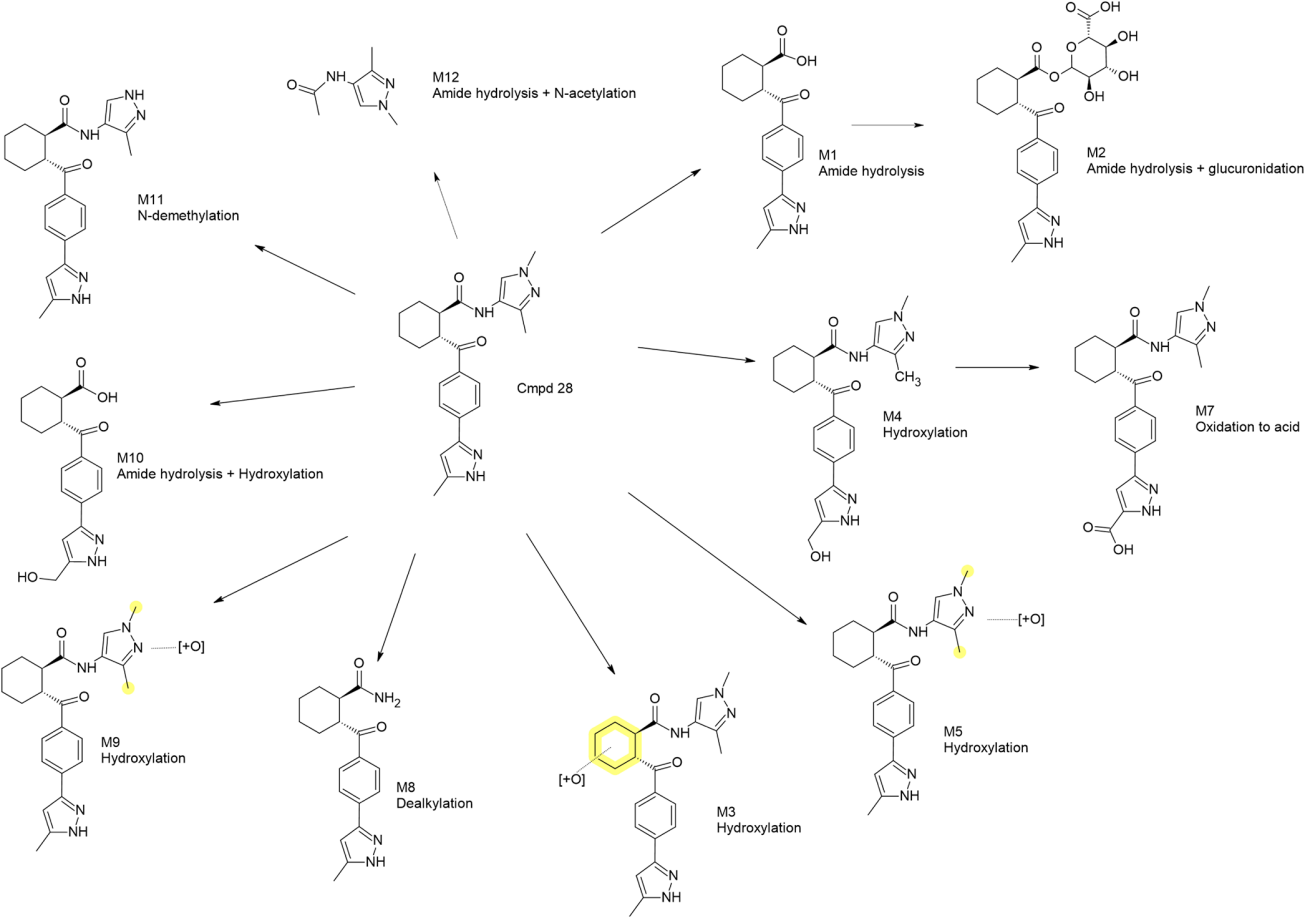
Metabolite profiling of Cmpd 28. Shown is a MetID scheme of Cmpd 28 together with its detected metabolites. Each metabolite is characterized by the observed transformation mechanism and the highlighted portion of the molecule where biotransformation takes place (in cases where exact conversion cannot be confidently assigned) or the exact chemical structure (where biotransformation assignment is certain). Detailed information about the species/system in which each metabolite is formed is found in Table 2.

The assessment of Cmpd 28 in rat hepatocytes gives a somewhat different metabolic profile compared to that of the dog, with no detection of the amide hydrolysis metabolites M1 and M2. The major metabolite in rat hepatocytes was the secondary metabolite M7. Cmpd 28 was assessed in both male and female rats *in vivo*, but, in contrast to Cmpd 16, no major differences were observed between genders. The major *in vivo* circulating metabolite was M1 (amide hydrolysis), which was not observed in the rat hepatocytes but was also a major metabolite in the dog (both *in vitro* and *in vivo*). The major rat *in vitro* metabolite, M7, was not seen in rat plasma, nor was M4, the precursor of M7. On the other hand, M4 and/or M7 were detected in the dog (both *in vitro* and *in vivo*). It is possible that M4 and/or M7 were also formed *in vivo* in the rat but eliminated via the bile and, therefore, not detected in the plasma samples.

Cmpd 28 was observed as two or three distinct peaks in hepatocyte incubations (dog and rat) as well as in rat plasma, whereas only a single peak was detected in dog plasma (Table 2). These multiple peaks likely correspond to different isomeric forms of the compound. The variation in the number of detected peaks may be attributed to differences in isomeric purity between batches, leading to inconsistent representation of isomers in various samples. Alternatively, it is possible that one or more isomers were present below the limit of detection in certain matrices, resulting in fewer observable peaks.

*Cmpds 47, 50, 51, and 55* were structurally similar and were all assessed *in vitro* and *in vivo* in dogs. Qualitatively, the metabolite IVIVc in dog was good for all compounds. The three observed metabolic pathways were glucuronidation, ring-opening of the azetidine, and *N*-oxidation (Figure 9). Metabolites from all three pathways were observed in hepatocytes for all compounds except Cmpd 55, for which no *N*-oxidation was seen (Table 3). On the other hand, *N*-oxidation was not observed in dog plasma or urine for Cmpd 55 either; hence, the metabolic pattern in hepatocytes was representative of *in vivo* biotransformation pathways. For Cmpd 51, *N*-oxide was detected at low levels in dog hepatocytes, but it was not observed in dog plasma or urine. No metabolites of Cmpd 50 were detected in plasma, but the two major *in vitro* metabolites (glucuronidation and ring-opening of the azetidine) were detected in dog urine (Table 3).

**Figure 9:**
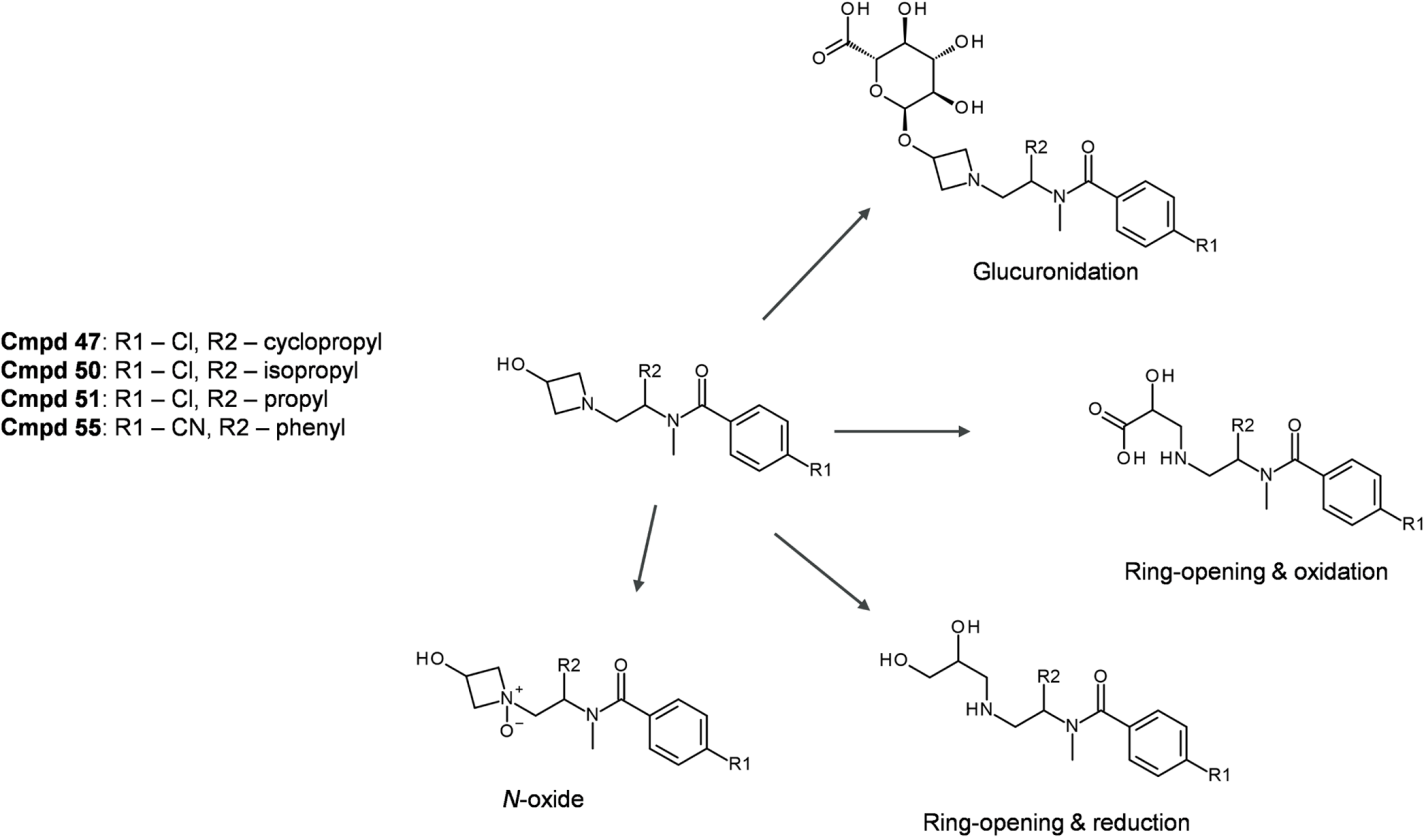
Metabolite profiles of Cmpd 47, Cmpd 50, Cmpd 51, and Cmpd 55. Each metabolite is characterized by the observed transformation mechanism and the chemical structure. Detailed information about the species/system in which each metabolite is formed is found in Table 3.

For most of the compounds, the conversion was considerably higher *in vivo* than *in vitro*. This is especially true for Cmpd 47, where the parent peak area was only 5% of the total (parent and metabolites) peak area detected in plasma after 30 min, whereas the parent peak area accounted for 83% in the hepatocytes after a 120 min incubation.

### Outlook—Extraction of MetID Information for ML/AI Applications

To enable early assessment of metabolite formation and metabolic pathways, reliable computational approaches for predicting drug metabolism are crucial. Many of these methods rely on ML, which requires large, high-quality data sets for accurate predictions.

As demonstrated herein, MetID data are commonly presented in schemes or figures, which are not readily machine readable. Furthermore, the schemes are based on the interpretation of MS data, which are not always conclusive, leading to Markush structures being reported. Although supported by scientific software, the data interpretation still relies heavily on the scientist’s knowledge and experience, as well as on whether the compound belongs to a well-known series or represents a novel structure.

Therefore, the data must undergo a process of conversion, interpretation, and curation by experts before it becomes usable for developing *in silico* approaches. This process often involves:

- Converting measured biotransformation data into parent-metabolite pairs for metabolite structure^25^ and metabolic pathway prediction.^54^
- Annotating metabolic reaction or pathway to support metabolic reaction or pathway class prediction.^55^
- Identifying and labeling SoMs in parent compounds for SoM prediction.^21^

Compounds should be represented in machine-readable formats such as SMILES or Structure Data Format (SDF), and parent–metabolite relationships should be defined in formats such as reaction SMILES or reaction data format (RDF). Specific to SoMs, the atom position annotations in the chemical structures should be preserved. However, manual conversion and annotation of these data are time-consuming and require domain expertise, especially if metabolites are depicted with a Markush structure, i.e., not absolutely defined. Several computational methods have been developed to automate or prioritize data extraction and annotation and can potentially be used in data curation. These include:

- Optical chemical structure recognition (OCSR): Chemical information from MetID data in PDF format can be converted into machine-readable formats by using OCSR tools. For example, DECIMER^56^ and OSRA^57^ extract chemical structures from images while ReactionDataExtractor,^58^ and RxnScribe^59^ are designed to extract data from reaction schemes.
- Atom-to-atom mapping (AAM): In the context of metabolism, AAM establishes a correspondence between atoms in parent compounds and their metabolites, enabling the automated identification of SoMs. Several publicly and commercially available AAM tools, such as AutoMapper from ChemAxon,^60^ RXNMapper,^61^ and Reaction Decoder Tool,^62^ are widely used to support chemical reaction analysis and prediction tasks.
- Annotation prioritization: Active learning approaches can optimize data measurement and annotation efforts. For example, a recent simulation study demonstrated that using active learning significantly reduces the annotation workload for SoM data while maintaining predictive accuracy.^23^

Work on integrating these methods into ML pipelines is ongoing. It is expected that ML frameworks will increase the accuracy and efficiency of metabolism prediction, thus reducing reliance on manual curation and enabling large-scale predictive modeling. Further advancements in data standardization, automation, and AI-driven feature extraction will continue to improve the usability of MetID data for ML applications.

## Conclusions

Here, we report metabolite identification data for 120 compounds derived from human hepatocyte incubations in the AstraZeneca data warehouse. The data were generated following routine procedures with the aim of understanding the metabolic liabilities of specific compounds and their respective compound series. The data set showed a good chemical space overlap with other project compounds evaluated in internal MetID experiments (*Other MetID compounds*), as well as an *in vitro* property distribution representative of the internal reference data set. In addition, the data set includes metabolites resulting from different reaction mechanisms, covering both phase I and phase II metabolism, thus making it a comprehensive data set. Note that only for a few metabolites the principal metabolic pathway could not be defined. However, about 40% of the described metabolites contained a Markush structure, i.e., the exact SoM was not identified due to challenges in mass spectrometry, such as poor MS response or the formation of MS fragments that were not specific enough to allow for the assignment of the exact SoM.

Using the MMP formalism, we also demonstrated how differences in MetID outcomes could be explained using observed chemical transformations that resulted in either a change in physicochemical properties or the elimination of certain metabolic pathway mechanisms via functional group protection. Metabolic stability was confirmed to correlate with lipophilicity, highlighting its importance in medicinal chemistry design. Careful modification of chemical moieties can fine-tune metabolic pathways, potentially leading to safer molecules by avoiding the formation of reactive metabolites. For selected compounds, where *in vivo* data were available, we investigated to what extent metabolites identified *in vitro* in hepatocytes corresponded to metabolites identified *in vivo* in rats or dogs. The conclusion, based on this data set, was that the *in vitro* to *in vivo* correlation was satisfactory, i.e., major metabolic pathways *in vivo* were captured in the *in vitro* system. However, differences in metabolite profiles exist between *in vitro* and *in vivo* results, among different species, and between males and females of the same species.

Finally, we describe how MetID data could be utilized further to develop tools to predict SoMs or likely metabolite structures, particularly using ML methods. MetID data require conversion and annotation to be useful. Automation tools can help streamline this process, improving prediction accuracy and efficiency. The integration of established and emerging software tools, coupled with effective data sharing and management strategies, will significantly enhance the metabolite identification landscape, leading to safer and more effective drug development. Therefore, we are making our set of 120 compounds with proprietary MetID schemes freely available as an open-access deposition on the Zenodo platform.^63^ In addition, we manually annotated the SoMs from these MetID schemes, characterized the corresponding atom environments, and explored their contribution to machine learning modeling as described in part 2.^64^ We hope to inspire other scientists and organizations to share additional proprietary metabolite data to increase knowledge about metabolism in the public domain and thereby enable even further improvements in metabolism prediction tools.

## Supplementary Material



## Data Availability

Metabolite identification schemes for 120 compounds discussed herein are available free of charge.^63^

## Notes

Table S1: List of monitored biotransformations; Table S2: SMILES of 120 *MetID* compounds) (PDF)

## References

[1] Mak K.-K., Epemolu O., Pichika M. R. (2022). The Role
of DMPK Science in Improving
Pharmaceutical Research and Development Efficiency. Drug Discovery Today.

[2] Kumar G. N., Surapaneni S. (2001). Role of Drug Metabolism in Drug Discovery
and Development. Med. Res. Rev..

[3] Benedetti M. S., Whomsley R., Poggesi I., Cawello W., Mathy F.-X., Delporte M.-L., Papeleu P., Watelet J.-B. (2009). Drug Metabolism
and Pharmacokinetics. Drug Metab. Rev..

[4] Zhang D., Luo G., Ding X., Lu C. (2012). Preclinical Experimental Models of
Drug Metabolism and Disposition in Drug Discovery and Development. Acta Pharm. Sin. B.

[5] Shu Y.-Z., Johnson B. M., Yang T. J. (2008). Role of Biotransformation
Studies
in Minimizing Metabolism-Related Liabilities in Drug Discovery. AAPS J..

[6] Kirchmair J., Göller A. H., Lang D., Kunze J., Testa B., Wilson I. D., Glen R. C., Schneider G. (2015). Predicting
Drug Metabolism: Experiment and/or Computation?. Nat. Rev. Drug Discovery.

[7] Lin J. H., Lu A. Y. H. (1997). Role of Pharmacokinetics and Metabolism in Drug Discovery
and Development. Pharmacol. Rev..

[8] Thompson R. A., Isin E. M., Ogese M. O., Mettetal J. T., Williams D. P. (2016). Reactive
Metabolites: Current and Emerging Risk and Hazard Assessments. Chem. Res. Toxicol..

[9] Zanger U. M., Schwab M. (2013). Cytochrome P450 Enzymes
in Drug Metabolism: Regulation
of Gene Expression, Enzyme Activities, and Impact of Genetic Variation. Pharmacol. Ther..

[10] Obach R. S. (2013). Pharmacologically
Active Drug Metabolites: Impact on Drug Discovery and Pharmacotherapy. Pharmacol. Rev..

[11] Hatsis P., Waters N. J., Argikar U. A. (2017). Implications for Metabolite Quantification
by Mass Spectrometry in the Absence of Authentic Standards. Drug Metab. Dispos..

[12] Blanz J., Williams G., Dayer J., Délémonté T., Gertsch W., Ramstein P., Aichholz R., Trunzer M., Pearson D. (2017). Evaluation of Relative MS Response Factors of Drug
Metabolites for Semi-Quantitative Assessment of Chemical Liabilities
in Drug Discovery. J. Mass Spectrom..

[13] MetaboLynx. https://www.waters.com/nextgen/be/en/library/application-notes/2006/automated-high-throughput-uplc-ms-ms-metabolite-id-using-metabolynx.html. (accessed 31 March 2025).

[14] Compound Discoverer. https://www.thermofisher.com/se/en/home/industrial/mass-spectrometry/liquid-chromatography-mass-spectrometry-lc-ms/lc-ms-software/multi-omics-data-analysis/compound-discoverer-software.html?erpType=Global_E1. (Accessed 31 March 2025).

[15] MetabolitePilot. https://sciex.com/cl/products/software/metabolitepilot-software. (Accessed 31 March 2025).

[16] MetaboScape. https://www.bruker.com/en/products-and-solutions/mass-spectrometry/ms-software/metaboscape.html. (Accessed 31 March 2025).

[17] MassMetaSite. https://mass-analytica.com/products/massmetasite/. (Accessed 31 March 2025).

[18] Bonn B., Leandersson C., Fontaine F., Zamora I. (2010). Enhanced Metabolite
Identification with MSE and a Semi-Automated Software for Structural
Elucidation. Rapid Commun. Mass Spectrom..

[19] Zelesky V., Schneider R., Janiszewski J., Zamora I., Ferguson J., Troutman M. (2013). Software Automation
Tools for Increased Throughput
Metabolic Soft-Spot Identification in Early Drug Discovery. Bioanalysis.

[20] Ahlqvist M., Leandersson C., Hayes M. A., Zamora I., Thompson R. A. (2015). Software-Aided
Structural Elucidation in Drug Discovery. Rapid
Commun. Mass Spectrom..

[21] Šícho M., Stork C., Mazzolari A., de Bruyn Kops C, Pedretti A., Testa B., Vistoli G., Svozil D., Kirchmair J. (2019). FAME 3: Predicting the Sites of Metabolism in Synthetic
Compounds and Natural Products for Phase 1 and Phase 2 Metabolic Enzymes. J. Chem. Inf. Model..

[22] Zaretzki J., Matlock M., Swamidass S. J. (2013). XenoSite: Accurately Predicting CYP-Mediated
Sites of Metabolism with Neural Networks. J.
Chem. Inf. Model..

[23] Chen Y., Seidel T., Jacob R. A., Hirte S., Mazzolari A., Pedretti A., Vistoli G., Langer T., Miljković F., Kirchmair J. (2024). Active Learning Approach for Guiding Site-of-Metabolism
Measurement and Annotation. J. Chem. Inf. Model..

[24] Djoumbou-Feunang Y., Fiamoncini J., Gil-de-la-Fuente A., Greiner R., Manach C., Wishart D. S. (2019). BioTransformer:
A Comprehensive Computational Tool
for Small Molecule Metabolism Prediction and Metabolite Identification. J. Cheminf..

[25] de
Bruyn Kops C., Šícho M., Mazzolari A., Kirchmair J. (2020). GLORYx: Prediction of the Metabolites Resulting from
Phase 1 and Phase 2 Biotransformations of Xenobiotics. Chem. Res. Toxicol..

[26] Meteor Nexus. Meteor Nexus highlights. https://www.lhasalimited.org/solutions/metabolite-identification-and-analysis/#Meteor. (Accessed 31 March 2025).

[27] Jung Y., Geng C., Bonvin A. M. J. J., Xue L. C., Honavar V. G. (2023). MetaScore:
A Novel Machine-Learning-Based Approach to Improve Traditional Scoring
Functions for Scoring Protein–Protein Docking Conformations. Biomolecules.

[28] Rydberg P., Gloriam D. E., Zaretzki J., Breneman C., Olsen L. (2010). SMARTCyp:
A 2D Method for Prediction of Cytochrome P450-Mediated Drug Metabolism. ACS Med. Chem. Lett..

[29] Afzelius L., Hasselgren Arnby C., Broo A., Carlsson L., Isaksson C., Jurva U., Kjellander B., Kolmodin K., Nilsson K., Raubacher F. (2007). State-of-the-art Tools for Computational Site
of Metabolism Predictions: Comparative Analysis, Mechanistical Insights,
and Future Applications. Drug Metab. Rev..

[30] Li J., Schneebeli S. T., Bylund J., Farid R., Friesner R. A. (2011). IDSite:
An Accurate Approach to Predict P450-Mediated Drug Metabolism. J. Chem. Theory Comput..

[31] Cruciani G., Carosati E., De Boeck B., Ethirajulu K., Mackie C., Howe T., Vianello R. (2005). MetaSite: Understanding
Metabolism in Human Cytochromes from the Perspective of the Chemist. J. Med. Chem..

[32] Miljković F., Medina-Franco J. L. (2024). Artificial Intelligence-Open Science Symbiosis in Chemoinformatics. Artif. Intell. Life Sci..

[33] ONIRO Oniro - The web platform for drug discovery. https://www.moldiscovery.com/software/oniro/. (Accessed 31 March 2025).

[34] Wenlock M. C., Potter T., Barton P., Austin R. P. (2011). A Method for Measuring
the Lipophilicity of Compounds in Mixtures of 10. J. Biomol. Screening.

[35] Wan H., Holmen A. G. (2009). High Throughput Screening of Physicochemical Properties
and In Vitro ADME Profiling in Drug Discovery. Comb. Chem. High Throughput Screening.

[36] Sohlenius-Sternbeck A.-K., Afzelius L., Prusis P., Neelissen J., Hoogstraate J., Johansson J., Floby E., Bengtsson A., Gissberg O., Sternbeck J., Petersson C. (2010). Evaluation
of The Human Prediction of Clearance from Hepatocyte and Microsome
Intrinsic clearance for 52 Drug Compounds. Xenobiotica.

[37] Temesi D. G., Martin S., Smith R., Jones C., Middleton B. (2010). High-Throughput
Metabolic Stability Studies in Drug Discovery by Orthogonal Acceleration
Time-of-Flight (OATOF) with Analogue-to-Digital Signal Capture (ADC). Rapid Commun. Mass Spectrom..

[38] Wan H., Bergström F. (2007). High Throughput
Screening of Drug-Protein Binding in
Drug Discovery. J. Liq. Chromatogr. Relat. Technol..

[39] Waters N. J., Jones R., Williams G., Sohal B. (2008). Validation of a Rapid
Equilibrium Dialysis Approach for the Measurement of Plasma Protein
Binding. J. Pharm. Sci..

[40] Austin R. P., Barton P., Mohmed S., Riley R. J. (2005). The Binding of Drugs
to Hepatocytes and its Relationship to Physicochemical Properties. Drug Metab. Dispos..

[41] RDKit. RDKit: Open-Source Cheminformatics Software. https://www.rdkit.org/. (Accessed 31 March, 2025).

[42] McInnes L., Healy J., Saul N., Großberger L. (2018). UMAP: Uniform
Manifold Approximation and Projection. J. Open
Source Softw..

[43] GitHub GitHub - lmcinnes/umap: Uniform Manifold Approximation and Projection. https://github.com/lmcinnes/umap. (31 March 2025).

[44] Bemis G. W., Murcko M. A. (1996). The Properties of
Known Drugs. 1. Molecular Frameworks. J. Med.
Chem..

[45] Rawden H.
C., Kokwaro G. O., Ward S. A., Edwards G. (2000). Relative Contribution
of Cytochromes P-450 and Flavin-Containing Monoxygenases to The Metabolism
of Albendazole by Human Liver Microsomes. Br.
J. Clin. Pharmacol..

[46] Wu Z., Lee D., Joo J., Shin J.-H., Kang W., Oh S., Lee D. Y., Lee S.-J., Yea S. S., Lee H. S., Lee T., Liu K.-H. (2013). CYP2J2 and CYP2C19 Are the Major Enzymes Responsible
for Metabolism of Albendazole and Fenbendazole in Human Liver Microsomes
and Recombinant P450 Assay Systems. Antimicrob.
Agents Chemother..

[47] Lutz J. D., Isoherranen N. (2012). Prediction of Relative In Vivo Metabolite Exposure
from In Vitro Data Using Two Model Drugs: Dextromethorphan and Omeprazole. Drug Metab. Dispos..

[48] Yu A., Haining R. L. (2001). Comparative Contribution to Dextromethorphan Metabolism
by Cytochrome P450 Isoforms in Vitro: Can Dextromethorphan Be Used
as a Dual Probe for Both CYP2D6 and CYP3A Activities?. Drug Metab. Dispos..

[49] Lipinski C. A., Lombardo F., Dominy B. W., Feeney P. J. (1997). Experimental and
Computational Approaches to Estimate Solubility and Permeability in
Drug Discovery and Development Settings. Adv.
Drug Delivery Rev..

[50] Wenlock M. C., Austin R. P., Barton P., Davis A. M., Leeson P. D. (2003). A Comparison
of Physiochemical Property Profiles of Development and Marketed Oral
Drugs. J. Med. Chem..

[51] Evans W. E., Relling M. V. (1999). Pharmacogenomics:
Translating Functional Genomics into
Rational Therapeutics. Science.

[52] Wassermann A. M., Dimova D., Iyer P., Bajorath J. (2012). Advances in Computational
Medicinal Chemistry: Matched Molecular Pair Analysis. Drug Dev. Res..

[53] Kramer C., Ting A., Zheng H., Hert J., Schindler T., Stahl M., Robb G., Crawford J. J., Blaney J., Montague S. (2017). Learning Medicinal Chemistry Absorption, Distribution,
Metabolism, Excretion, and Toxicity (ADMET) Rules from Cross-Company
Matched Molecular Pairs Analysis (MMPA). J.
Med. Chem..

[54] Shah H. A., Liu J., Yang Z., Feng J. (2021). Review of Machine Learning Methods
for the Prediction and Reconstruction of Metabolic Pathways. Front. Mol. Biosci..

[55] Baranwal M., Magner A., Elvati P., Saldinger J., Violi A., Hero A. O. (2020). A Deep Learning Architecture for
Metabolic Pathway Prediction. Bioinformatics.

[56] Rajan K., Brinkhaus H. O., Agea M. I., Zielesny A., Steinbeck C. (2023). DECIMER.ai:
An Open Platform for Automated Optical Chemical Structure Identification
Segmentation And Recognition In Scientific Publications. Nature Communications.

[57] Filippov I. V., Nicklaus M. C. (2009). Optical Structure
Recognition Software To Recover Chemical
Information: OSRA, An Open Source Solution. J. Chem. Inf. Model..

[58] Wilary D. M., Cole J. M. (2023). ReactionDataExtractor 2.0: A Deep Learning Approach
for Data Extraction from Chemical Reaction Schemes. J. Chem. Inf. Model..

[59] Qian Y., Guo J., Tu Z., Coley C. W., Barzilay R. (2023). RxnScribe: A Sequence
Generation Model for Reaction Diagram Parsing. J. Chem. Inf. Model..

[60] AutoMapper. https://dl.chemaxon.com/docs/HTML/docs17210/AutoMapper.html. (Accessed 31 March 2025).

[61] Schwaller P., Hoover B., Reymond J.-L., Strobelt H., Laino T. (2021). Extraction
of Organic Chemistry Grammar from Unsupervised Learning of Chemical
Reactions. Sci. Adv..

[62] Rahman S. A., Torrance G., Baldacci L., Martínez
Cuesta S., Fenninger F., Gopal N., Choudhary S., May J. W., Holliday G. L., Steinbeck C., Thornton J. M. (2016). Reaction Decoder Tool (RDT): Extracting Features from
Chemical Reactions. Bioinformatics.

[63] Ahlqvist, M. ; Bonner Karlsson, I. ; Ekdahl, A. ; Ericsson, C. ; Jurva, U. ; Miljković, F. ; Chen, Y. ; Winiwarter, S. AstraZeneca metabolite identification (MetID) schemes for 120 compounds; Zenodo, 2025 10.5281/zenodo.15319258. PMC12587390 41085376

[64] Chen, Y. ; Winiwarter, S. ; Jacob, R. A. ; Ahlqvist, M. ; Mazzolari, A. ; Miljković, F. ; Kirchmair, J. Metabolite Identification Data in Drug Discovery, Part 2: Site-of-Metabolism Annotation, Analysis, and Exploration for Machine Learning. Mol. Pharmaceutics 2025, 10.1021/acs.molpharmaceut.5c00740. PMC12587399 41117345

