# Supplementary material for: Metabolite Identification Data in Drug Discovery, Part 1: Data Generation and Trend Analysis
Source: Mol Pharm. 2025 Oct 14;22(11):6788–802. doi: 10.1021/acs.molpharmaceut.5c00738 (PMC12587390; doi:10.1021/acs.molpharmaceut.5c00738)
Supplement: Supplementary file 1 [file mp5c00738_si_001.pdf]

Supporting information

Metabolite Identification Data in Drug Discovery, Part 1: Data Generation and Trend Analysis

Marie Ahlqvist,[1] Isabella Bonner Karlsson,[1] Anja Ekdahl,[1] Cecilia Ericsson,[1] Ulrik Jurva,[1] Filip Miljković,[2] Ya Chen,[2,3] and Susanne Winiwarter[1]

[1]Drug Metabolism and Pharmacokinetics, Research and Early Development, Cardiovascular, Renal and Metabolism (CVRM), BioPharmaceuticals R&D, AstraZeneca, 431 83 Mölndal, Sweden

[2]Medicinal Chemistry, Research and Early Development, Cardiovascular, Renal and Metabolism (CVRM), BioPharmaceuticals R&D, AstraZeneca, 43183 Mölndal, Sweden

[3]Department of Pharmaceutical Sciences, Division of Pharmaceutical Chemistry, Faculty of Life Sciences, University of Vienna, 1090 Vienna, Austria

Content

Table S1. List of Monitored Biotransformations

Table S2: SMILES of 120 MetID Compounds

Table S1. List of Monitored Biotransformations

Shift in m/z	Chemical formula change	Transformation	Phase
0		Parent	0
2.0157	H2	Reduction	1
11.9636	+O-H4	Hydroxylation + 2 x Desaturation	1
13.9793	+O-H2	Hydroxylation + Desaturation	1
14.0157	+CH2	Methylation	2
15.9949	+O	Hydroxylation	1
18.0106	+H2O	Hydrolysis	1
27.9585	+O2-H4	2 x Hydroxylation + 2 x Desaturations	1
29.9742	+O2-H2	2 x Hydroxylation + Desaturation	1
31.9898	+O2	2 x Hydroxylation	1
34.0055	+H2O2	Hydroxylation + Hydrolysis	1
42.0106	+C2H2O	Acetylation	2
57.0215	+C2H3NO	Glycine conjugation	2
79.9568	+SO3	Sulfate conjugation	2
95.9517	+O4S	Hydroxylation + Sulfate conjugation	2
107.0041	+C2H5NO2S	Taurine conjugation	2
119.0041	+C3H5NO2S	Cysteine	2
132.0423	+C5H8O4	Ribose	1
143.0946	+C7H13NO2	Carnitine conjugation	1
161.0147	+C5H7NO3S	N-acetylcysteine	2
162.0528	+C6H10O5	Glucose	1
174.0164	+C6H6O6	Desaturation + Glucuronide	2

176.0256	+C5H8N2O3S	Cysteinylglycine	1
176.0321	+C6H8O6	Glucuronide	2
192.027	+C6H8O7	Hydroxylation + Glucuronide	2
203.0794	+C8H13NO5	N-acetylglucosamine	1
208.0219	+C6H8O8	2 x Hydroxylation + Glucuronide conjugation	2
305.0682	+C10H15N3O6S	Glutathione	2
321.0631	+C10H15N3O7S	Hydroxylation + Glutathione	2
323.0787	+C10H17N3O7S	Hydrolysis + Glutathione	2
-2.0157	-H2	Desaturation	1
-4.0313	-H4	2 x Desaturation	1
-14.0157	-CH2	Demethylation	1
-15.0109	-NH	Loss of NH	1
-15.9772	-S+O	Thioureas to Ureas	1
-18.0106	-H2O	Dehydration	1
-25.9793	+H2-CO	Reduction + Decarbonylation	1
-26.0157	-C2H2	Ring deethylation	1
-27.9949	-CO	Decarbonylation	1
-28.0313	-C2H4	Deethylation	1
-31.9721	-S	Loss of S	1
-32.9799	-SH	Loss of SH	1
-42.047	-C3H6	Isopropyl dealkylation	1

Table S2: SMILES of 120 MetID Compounds

Compound ID	SMILES
Cmpd 1	CCc1c(c2c(n1Cc3ccccc3)cccc2OCC(=O)O)C(=O)C(=O)N
Cmpd 2	CC(C)[C@H]1c2ccc(cc2CC[C@@]1(CCN(C)CCCc3[nH]c4ccccc4n3)OC(=O)COC)F
Cmpd 3	C[C@H](CO)Nc1cc(NS(C)(=O)=O)nc(SCc2cccc(F)c2F)n1
Cmpd 4	CC1=C2[C@H](C(=O)[C@@]3([C@H](C[C@@H]4[C@]([C@H]3[C@@H]([C@@](C2(C)C)(C[C@@H]1OC(=O)[C@@H]([C@H](c5ccccc5)NC(=O)c6ccccc6)O)O)OC(=O)c7ccccc7)(CO4)OC(=O)C)O)C)OC(=O)C
Cmpd 5	Cc1nc(NC(=N)N)sc1-c1cccc(NC(=O)c2ccccc2)c1
Cmpd 6	CCn1c(=O)oc2ccc(-c3ccc(C[C@@H](C#N)NC(=O)[C@@H]4CNCCCO4)cc3)cc21
Cmpd 7	Cc1c(sc(n1)NC(=N)N)c2cccc(c2)NC(=O)c3ccc(cc3)F
Cmpd 8	C[C@H](CO)Oc1nc(SCc2cccc(F)c2F)nc2nc(N)sc12
Cmpd 9	CC(C)C[C@H](CO)Nc1c2c([nH]c(=O)s2)nc(n1)SC(C)c3ccccn3
Cmpd 10	O=C(Nc1cnn2cccnc12)[C@@H]1CCCC[C@H]1C(=O)c1ccc(-c2cc[nH]n2)cc1
Cmpd 11	Cn1cc(NC(=O)C2CCCCC2C(=O)c2ccc(-c3cc[nH]n3)cc2)c(C(N)=O)n1
Cmpd 12	Nc1nnc(-c2ccc(C3(c4ccc(OCc5ccccn5)cc4)CCCO3)cn2)o1
Cmpd 13	c1ccnc(c1)COc2ccc(cc2)C3(CCCO3)c4ccc(nc4)c5nnc(o5)N
Cmpd 14	Cc1c-2c(on1)COc3cccc(c3)CCCS(=O)(=O)NCc4ccc2cc4
Cmpd 15	Nc1cnc(-c2ccc(C3(c4ccc(-c5nnco5)nc4)CCCO3)cc2)cn1
Cmpd 16	Cn1cc(NC(=O)[C@@H]2CCCC[C@H]2C(=O)c2ccc(-c3cc[nH]n3)cc2)c(C(F)(F)F)n1
Cmpd 17	COc1cc(NC(=O)[C@@H]2CCCC[C@H]2C(=O)c2ccc(-c3cc[nH]n3)cc2)ncn1
Cmpd 18	Cn1ncc(NC(=O)[C@@H]2CCCC[C@H]2C(=O)c2ccc(-c3cc[nH]n3)cc2)c1C(N)=O
Cmpd 19	Cc1cc(-c2ccc(C(=O)[C@@H]3CCCC[C@H]3C(=O)Nc3c(C)nn(C)c3C(N)=O)cc2)n[nH]1
Cmpd 20	Cc1cc(-c2ccc(C(=O)C3CCCCC3C(=O)Nc3cnn(C)c3C(N)=O)c(F)c2)n[nH]1
Cmpd 21	Cn1ncc(NC(=O)[C@@H]2C[C@@H]3C[C@@H]3C[C@H]2C(=O)c2ccc(-c3cc[nH]n3)cc2)c1C(F)(F)F
Cmpd 22	Cn1cc(NC(=O)[C@@H]2C[C@@H]3C[C@@H]3C[C@H]2C(=O)c2ccc(-c3cc[nH]n3)cc2)c(C(F)(F)F)n1
Cmpd 23	Cc1cc(-c2ccc(C(=O)[C@@H]3CCCC[C@H]3C(=O)Nc3cn(C)nc3C(F)F)cc2)n[nH]1
Cmpd 24	Cc1cc(-c2ccc3c([C@@H]4CCCC[C@H]4C(=O)Nc4cn(C)nc4C(N)=O)noc3c2)n[nH]1
Cmpd 25	Cn1ncc(NC(=O)[C@@H]2CCCC[C@H]2C(=O)c2ccc(-c3cc[nH]n3)cc2)c1C(F)(F)F
Cmpd 26	Cc1cc(-c2ccc(C(=O)[C@@H]3CCCC[C@H]3C(=O)Nc3cnn4c3OCC4)cc2)n[nH]1
Cmpd 27	Cn1cc(NC(=O)[C@@H]2CCCC[C@H]2C(=O)c2ccc(-c3cc[nH]n3)cc2)c(C(F)F)n1
Cmpd 28	Cc1cc(-c2ccc(C(=O)[C@@H]3CCCC[C@H]3C(=O)Nc3cn(C)nc3C)cc2)n[nH]1
Cmpd 29	O=C1NCCn2ncc(NC(=O)[C@@H]3CCCC[C@H]3C(=O)c3ccc(-c4ccn[nH]4)cc3)c21
Cmpd 30	Cn1c(cc2c1cnc(c2)Br)C(=O)N3CCc4cc(ccc4C3)CN(C)C
Cmpd 31	Cc1ccc(-n2nc(C)c3cnc(-c4ccc([C@H]5C[C@@H]5C(=O)O)cc4F)cc32)nc1
Cmpd 32	Cc1ccc(-n2nc(C)c3ccc(-c4ccc([C@H]5C[C@@H]5C(=O)O)cc4)cc32)cc1
Cmpd 33	O=S1(=O)c2ccccc2CN1c1cncc(Oc2cccnc2)n1

Cmpd 34	CCOc1cc(c(cc1N2CCOCC2)c3ccc(c(=O)[nH]3)c4nc5c(s4)CN(CC5)C(=O)COC)Cl
Cmpd 35	CCOc1cc(c(cc1N2CCOCC2)C(=O)Nc3nc4ccc(cc4s3)S(=O)(=O)CCCN5CCN(CC5)C)Cl
Cmpd 36	O=C(N[C@H]1CN(S(=O)(=O)c2ccccc2)C[C@@H]1C(=O)N[C@H]1CCCNC1)c1cc(-c2ccccc2)on1
Cmpd 37	CC(C)(C(=O)N[C@H](COCc1ccccc1)C(=O)N2CCC3=NN(C(=O)[C@@]3(C2)Cc4ccccc4)C)N
Cmpd 38	CCOc1cc(c(cc1N2CCOCC2)C(=O)NC(=O)Nc3nc4ccc(cc4s3)CN5CCN(CC5)C)Cl
Cmpd 39	CN1CCN(CC1)CCCS(=O)(=O)c2ccc3c(c2)sc(n3)NC(=O)NC(=O)c4ccccc4Cl
Cmpd 40	CC(C)(C)NC(=O)NCCN1CCC(CC1)(CNC(=O)c2cc(cc(c2)Cl)Cl)O
Cmpd 41	CC(C)(C)NC(=O)NCCN1CCC(CC1)(CNC(=O)c2cc(cc(c2)Cl)Cl)CO
Cmpd 42	CCC[C@@H](CN1CC(O)C1)N(C)C(=O)c1ccc(F)cc1
Cmpd 43	CN(C(=O)c1ccc(F)c(C(F)(F)F)c1)[C@H](CN1CC(O)C1)C1CC1
Cmpd 44	Cc1cc(C(=O)N(C)[C@H](CN2CC(O)(C3CC3)C2)C2CC2)ccc1F
Cmpd 45	CCC[C@@H](CN1CC(O)C1)N(C)C(=O)c1ccc(F)c(C)c1
Cmpd 46	CO[C@H](C)[C@@H](CN1CCCC1)N(C)C(=O)c1ccc(F)c(C)c1
Cmpd 47	CN(C(=O)c1ccc(Cl)cc1)[C@H](CN1CC(O)C1)C1CC1
Cmpd 48	CN(C(=O)c1ccc(Br)cc1)[C@H](CN1CC(O)C1)C1CC1
Cmpd 49	CCC[C@@H](CN1CC(O)C1)N(C)C(=O)c1ccc(F)c(F)c1
Cmpd 50	CC(C)[C@@H](CN1CC(C1)O)N(C)C(=O)c2ccc(cc2)Cl
Cmpd 51	CCC[C@@H](CN1CC(O)C1)N(C)C(=O)c1ccc(Cl)cc1
Cmpd 52	CC(C)C[C@@H](CN1CC(O)C1)N(C)C(=O)c1ccc(F)c(F)c1
Cmpd 53	CC[C@H](C)[C@@H](CN1CC(O)C1)N(C)C(=O)c1ccc(C#N)cc1F
Cmpd 54	CN(C(=O)c1ccc(C#N)cc1)[C@H](CN1CC(O)C1)C1CCC1
Cmpd 55	CN(C(=O)c1ccc(C#N)cc1)[C@H](CN1CC(O)C1)c1ccccc1
Cmpd 56	CCC(=O)N(Cc1cc(C)nc2[nH]c(=O)oc12)c1cccc(F)c1
Cmpd 57	Cc1c(sc(n1)NC(=O)C)c2cc3c(c(c2)NS(=O)(=O)C)C(=O)N(C3)[C@@H](C)C4CC4
Cmpd 58	Cc1c(sc(n1)NC(=O)C)c2cc3c(c(c2)S(=O)(=O)NC)C(=O)N(C3)[C@@H](C)C4CC4
Cmpd 59	c1cc(c(c(c1)F)c2c(ccc(n2)C(=O)Nc3cnccc3N4CCC[C@@H](C4)N)F)F
Cmpd 60	Cc1c(cccn1)NC(=O)c2ccc3c(c2)CC[C@H]4[C@]3(CC[C@@](C4)(C(F)(F)F)O)Cc5ccccc5
Cmpd 61	CC1(C[C@](C=C(C1=O)C#N)(c2c(cc(cn2)F)C(F)F)OC)C
Cmpd 62	c1ccc(cc1)c2cccc3c2c(nc(n3)c4cc(cnc4)S(=O)(=O)N)NCc5ccccn5
Cmpd 63	COc1cc(N(CC(C)C)c2cc(C(F)(F)F)ccc2F)cnc1C(=O)[C@H]1C[C@@H]1C(=O)O
Cmpd 64	COc1ccc(-c2nnc(C(=O)N3CC(Oc4ccc(CN5CCC(C)(CO)C5)cc4C)C3)o2)cc1
Cmpd 65	COc1ccc(-c2nnc(C(=O)N3CC(Oc4ccc(CN5CCC(C)(O)C5)cc4)C3)o2)cc1
Cmpd 66	CC1(O)CCN(Cc2ccc(OC3CN(C(=O)c4nnc(-c5ccccc5)o4)C3)cc2)C1
Cmpd 67	COc1ccc(-c2nnc(C(=O)N3CC(Oc4ccc(CN(C)C)c(C)c4)C3)o2)cc1
Cmpd 68	COc1ccc(-c2nnc(C(=O)N3CC(Oc4ccc(CN5CCC(CO)CC5)cc4)C3)o2)cc1
Cmpd 69	COc1ccc(-c2nnc(C(=O)N3CC(Oc4ccc(CN5CCC(C)(CO)C5)cc4)C3)o2)cc1
Cmpd 70	COc1ccc(-c2nnc(C(=O)N3CC(Oc4ccc(CN5CCC(C)(CO)C5)cc4)C3)o2)cc1

Cmpd 71	COc1ccc(-c2nnc(C(=O)N3CC(Oc4ccc(CN5CCC(F)(CO)CC5)cc4)C3)o2)cc1
Cmpd 72	Cn1c2c(c3ccc(-n4ccc(OCc5ccc(F)cn5)cc4=O)cc31)CNCC2
Cmpd 73	COc1ccc(-c2nnc(C(=O)N3CC(Oc4ccc(CN5CCC6(CCOC6)C5)cc4)C3)o2)cc1
Cmpd 74	COc1ccc(-c2nnc(C(=O)N3CC(Oc4ccc(CN5CCC(C)(CO)CC5)cc4)C3)o2)cc1
Cmpd 75	COc1ccc(-c2nnc(C(=O)N3CC(Oc4ccc(CN5CCC(C)(CO)C5)cc4)C3)o2)cc1
Cmpd 76	COc1ccc(-c2nnc(C(=O)N3CC(Oc4ccc(CN5CCCC(C)(O)CC5)cc4)C3)o2)cc1
Cmpd 77	COc1ccc(-c2nnc(C(=O)N3CC(Oc4ccc(CN5CC6(CCCO6)C5)cc4)C3)o2)cc1
Cmpd 78	Cc1cc(OC2CN(C(=O)c3nnc(-c4ccccc4)o3)C2)ccc1CN1CCC(C)(CO)CC1
Cmpd 79	COc1ccc(-c2nnc(C(=O)N3CC(Oc4ccc(CN5CC6(COC6)C5)cc4C)C3)o2)cc1
Cmpd 80	FC(F)C1CN(C1)Cc2ccc(cc2)OC3CN(C3)C(=O)c4nnc(o4)-c5ccccc5
Cmpd 81	COc1ccc(-c2nnc(C(=O)N3CC(Oc4ccc(CN5CC(C)(O)C5)c(C)c4)C3)o2)cc1
Cmpd 82	Cc1cc(ccc1OC2CN(C2)C(=O)c3nnc(o3)c4ccc(cc4)OC)CN(C)C
Cmpd 83	O=c1cc(OCc2ccccc2)ccn1-c1ccc2c(cnn2CCN2CCCC2)c1
Cmpd 84	COc1ccc(cc1)c2nnc(o2)C(=O)N3CC(C3)Oc4ccc(cc4)CN5CC6(C5)CCOC6
Cmpd 85	COc1ccc(-c2nnc(C(=O)N3CC(Oc4ccc(CN5CCCC(O)CC5)cc4)C3)o2)cc1
Cmpd 86	N#Cc1ccc(N2N=C3c4ccc(C(=O)N5CCC(O)CC5)nc4CC[C@H]3[C@H]2C2CCCC2)cc1Cl
Cmpd 87	Cc1noc(C)c1-c1ccc(CNS(=O)(=O)c2c(C)nn(C)c2Cl)cc1
Cmpd 88	Cc1noc(C)c1-c1ccc(CNS(=O)(=O)c2ccccc2)cc1
Cmpd 89	CC1(C)Oc2cc(NS(C)(=O)=O)ccc2N(c2ccc(F)cc2)C1=O
Cmpd 90	c1ccc\2c(c1)COc3cc(ccc3/C2=C/c4ccc5c(c4)[nH]c(=O)n5[C@@H]6C[C@@H]7COCCN7C6)F
Cmpd 91	NC(=O)NC(=O)C(Nc1ccc2c(c1)CCC2)c1ccccc1
Cmpd 92	NC(=O)C[C@H]1COc2cc(Cl)ccc2N1C(=O)c1ccc2c(c1)NC(=O)CO2
Cmpd 93	NC(=O)CC1COc2cc(F)ccc2N1C(=O)c1ccc2c(c1)NC(=O)CO2
Cmpd 94	Cc1c(cn(c1c2ccccc2C(F)(F)F)CCO)C(=O)Nc3ccc(cc3)S(=O)(=O)C
Cmpd 95	C[C@]12C=CC(=O)C=C1CC[C@@H]1[C@@H]2[C@@H](O)C[C@@]2(C)[C@H]1CC[C@]2(O)C(=O)CO
Cmpd 96	O=C(c1ccc(-c2ccc(Cl)cc2)o1)N(Cc1ccccn1)c1ccc(N2CCNCC2)cc1
Cmpd 97	C[C@@H](Sc1nnc(-c2cn[nH]c(=O)c2)n1C)c1nnn(-c2cccc(Cl)c2)n1
Cmpd 98	C[C@H](c1nc(on1)c2cccc(c2)Cl)Oc3nnc(n3C)c4cc(=O)[nH]nc4
Cmpd 99	C[C@H]1CN(c2cc(N(CCO)Cc3cccc4ccccc34)nc(=O)[nH]2)CCO1
Cmpd 100	C[C@@H](Oc1ccccc1)c1cc(C(=O)N(C)CCO)cn2c(=O)cc(N3CCOCC3)nc12
Cmpd 101	CC(C)(C(=O)N[C@H](Cc1c[nH]c2c1cccc2)C(=O)N3CCC[C@](C3)(Cc4ccccc4)C(=O)N(C)N(C)C)N
Cmpd 102	CN1CCn2c(c(cn2)NC(=O)[C@@H]3CCCC[C@H]3C(=O)c4ccc(cc4)c5cc[nH]n5)C1=O
Cmpd 103	Cc1cc(-c2ccc(C(=O)[C@@H]3CCCC[C@H]3C(=O)Nc3cnn(C)c3C(N)=O)cc2)[nH]n1
Cmpd 104	Cc1cc(-c2ccc(C(=O)[C@@H]3CCCC[C@H]3C(=O)Nc3cnn(C)c3C(=O)NOC(C)(C)C)cc2)[nH]n1
Cmpd 105	COc1ccc(-c2nnc(C(=O)N3CC(Oc4ccc(CN5CC6(COC6)C5)c(C)c4)C3)o2)cc1
Cmpd 106	CNC(=O)CC1COc2ccccc2N1C(=O)c1ccc2c(c1)NC(=O)CO2

Cmpd 107	CNC(=O)CC1COc2cc(ccc2N1C(=O)c3ccc4c(c3)NC(=O)CO4)Br
Cmpd 108	CNC(=O)CC1COc2cc(Cl)ccc2N1C(=O)c1ccc2c(c1)NC(=O)CO2
Cmpd 109	Cc1cc(-c2ccc(C(=O)[C@@H]3CCCC[C@H]3C(=O)Nc3cnn(C)c3S(N)(=O)=O)cc2)n[nH]1
Cmpd 110	Cc1cc(n[nH]1)c2ccc(cc2)C(=O)[C@@H]3CCCC[C@H]3C(=O)Nc4cnn5c4C(=O)N(CC5)C
Cmpd 111	CCOc1ncc(C)c2c1[C@H](c1ccc(C#N)cc1OC)C(C(N)=O)=C(C)N2
Cmpd 112	Cc1c(c(n(n1)c2ccccc2)N)c3ccccc3
Cmpd 113	Cc1c(c(n(n1)c2ccccc2)N)c3cccc(c3)C(F)(F)F
Cmpd 114	CCc1c(c2c(n1Cc3ccccc3)cccc2OCC(=O)OC)C(=O)C(=O)N
Cmpd 115	COc1ccc(CCO[C@@H]2CCCC[C@H]2N2CC[C@@H](O)C2)cc1OC
Cmpd 116	c1cc(nc(c1)OCc2ccc(cc2F)C#N)N3CCN(C4C3CC4)Cc5nc6ccc(cc6n5C[C@@H]7CCO7)C(=O)O
Cmpd 117	CC1(c2ccc(Cl)cn2)Oc2cccc(C3CCN(Cc4nc5ccc(C(=O)O)cc5n4C[C@@H]4CCO4)CC3)c2O1
Cmpd 118	COc1cc(N2CCC(N3CCO[C@@H](CO)C3)CC2)ccc1Nc1ncc2sc(C(C)(C)O)c(OC(C)C)c2n1
Cmpd 119	CC(C)C(C)(c1ccc(cc1)c2ccc(nn2)C(C)(C)O)c3ccc(cn3)OCc4ccccn4
Cmpd 120	CC(C)C(C)(c1ccc(cc1)c2ccc(nn2)C(C)(C)O)c3ccc(cn3)OCc4ccccn4
